# Functional Divergence of *G* and Its Homologous Genes for Green Pigmentation in Soybean Seeds

**DOI:** 10.3389/fpls.2021.796981

**Published:** 2022-01-05

**Authors:** Yusuke Tokumitsu, Takuto Kozu, Hiroshi Yamatani, Takeshi Ito, Haruna Nakano, Ayaka Hase, Hiroki Sasada, Yoshitake Takada, Akito Kaga, Masao Ishimoto, Makoto Kusaba, Taiken Nakashima, Jun Abe, Tetsuya Yamada

**Affiliations:** ^1^Graduate School of Agriculture, Hokkaido University, Sapporo, Japan; ^2^Institute of Crop Science, National Agriculture and Food Research Organization, Tsukuba, Japan; ^3^Graduate School of Science, Hiroshima University, Higashihiroshima, Japan; ^4^Faculty of Agriculture, Hokkaido University, Sapporo, Japan; ^5^Western Region Agricultural Research Center, National Agriculture and Food Research Organization, Fukuyama, Japan

**Keywords:** *Glycine max*, chlorophyll, seed coat, wild soybean (*G. soja* Sieb. and Zucc.), landraces, domestication, yellow soybean

## Abstract

The degradation of chlorophyll in mature soybean seeds is closely related to the development of their yellow color. In this study, we examined *G*, its homologue *G-like* (*GL*), and their mutant alleles and investigated the relationship between these genes and chlorophyll accumulation in the seed coats of mature seeds. Transient expression of G and GL proteins fused with green fluorescent protein revealed that both were localized in plastids. Overexpression of *G* resulted in the accumulation of chlorophyll in the seed coats and cotyledons of mature seeds, indicating that high expression levels of *G* result in chlorophyll accumulation that exceeds its metabolism in the seeds of yellow soybean. Analysis of near isogenic lines at the *G* locus demonstrated a significant difference in the chlorophyll content of the seed coats and cotyledons of mature seeds when *G* and mutant *g* alleles were expressed in the *d_1_d_2_* stay-green genetic background, indicating that the G protein might repress the SGR-independent degradation of chlorophyll. We examined the distribution of mutant alleles at the *G* and *GL* loci among cultivated and wild soybean germplasm. The *g* allele was widely distributed in cultivated soybean germplasm, except for green seed coat soybean lines, all of which contained the *G* allele. The *gl* alleles were much fewer in number than the *g* alleles and were mainly distributed in the genetic resources of cultivated soybean from Japan. None of the landraces and breeding lines investigated in this study were observed to contain both the *g* and *gl* alleles. Therefore, in conclusion, the mutation of the *G* locus alone is essential for establishing yellow soybeans, which are major current soybean breeding lines.

## Introduction

Soybean (*Glycine max*, 2*n* = 2*x* = 40) is one of the most economically important crops worldwide because its seeds contain high-quality proteins and have an amino acid score comparable to that of beef and egg white. Soybean is used as a source of food and forage and is used in vegetable and industrial oils due to the high lipid content of the seeds (Li, 2004). The crop originated from its wild relative *G. soya*, which is native to East Asia, and was domesticated in China approximately 3,000–5,000 years ago (Fukuda, 1933; Nagata, 1959; Hymowitz and Newell, 1981). The agronomic traits of soybean have been refined continuously during the process of domestication from *G. soya* to cultivated soybean. Elucidating the molecular mechanisms of crop domestication provides a thorough understanding of the crop’s evolution and valuable information not only about crop improvement but also about the origins of agriculture (Jones and Brown, 2000). Recently, the molecular mechanisms of several domesticated traits in soybean have been elucidated, including environmental adaptability, lack of pod shattering, lack of hard seededness, increased seed size, and absence of vine growth habit (Liu et al., 2007; Dong et al., 2014; Sun et al., 2015; Wang et al., 2019).

**FIGURE 1:**
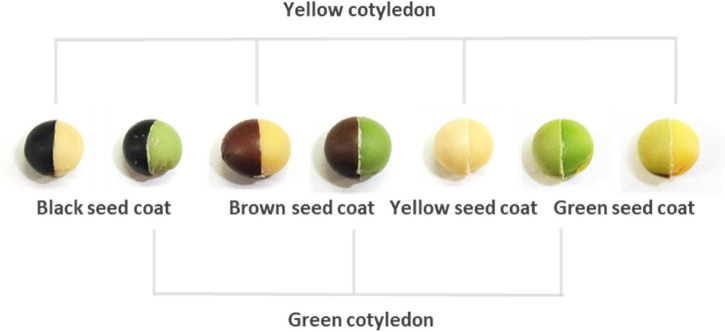
Representative seed coats and cotyledon colors in soybeans. The right half of each seed has the seed coat removed to expose the cotyledons.

During domestication, soybean’s adaptability to cultivation and palatability to humans resulted in wide genetic diversity for several traits in landraces (Zhou et al., 2015). Seed color represents one of these diversified traits. Most current breeding soybean cultivars exhibit a yellow color in the mature seeds, although various seed colors are found among soybean landraces. The seed colors of landraces are closely associated with various processing applications in soybean (Hwang et al., 2020) and are roughly divided into seed coat and cotyledon colors, but the seed coat color varies more than that of cotyledons (Figure 1). Black, brown, green, and yellow are predominantly recognized as seed coat colors; however, yellow seed coats are actually colorless, reflecting the color of the cotyledon. Clarifying the molecular mechanisms of seed coat pigmentation can provide a better understanding of how yellow soybeans are established, because they are thought to have been generated from landraces that exhibit various seed colors. The molecular mechanism underlying black, brown, and yellow pigmentation is well known. Four loci (*I*, *R*, *T*, and *W1*) were identified during investigations on the molecular mechanism of non-green seed coat pigmentation (Todd and Vodkin, 1993; Zabala and Vodkin, 2003, 2007; Senda et al., 2004; Gillman et al., 2011). Seed coat pigmentation is strictly determined by the epistasis of these genes, and their partial expression allows the spatial distribution of pigmentation, such as in the hilum or saddle-shaped region (Toda et al., 2012; Cho et al., 2017).

The molecular mechanism underlying green pigmentation in seed coats is complex because in mature plants, it can occur simultaneously with pigmentation in other tissues, including the cotyledon, or only in the seed coat (Figure 1). The former is caused by stay-green mutations caused by the *d*_1_ and *d*_2_ mutant alleles or the *cytG* allele. Two recessive alleles, *d*_1_ and *d*_2_, delay the degradation of chlorophyll in leaves, pod walls, seed coats, and cotyledons of mature soybean plants (Woodworth, 1921). The *d*_1_ and *d*_2_ loci are functionally redundant mutants of the *SGR* ortholog, which is known to be responsible for Mendel’s green cotyledon in pea (*Pisum sativum*) (Sato et al., 2007; Fang et al., 2014; Nakano et al., 2014). The stay-green mutation associated with *cytG* displays maternal inheritance behavior and delays the degradation of chlorophyll in the same variety of tissues as the *d*_1_ and *d*_2_ mutations (Terao, 1918). A 5-nucleotide insertion in the *cytG* locus, which is located in the chloroplast genome of soybean, causes a frameshift mutation in *PsbM*, which encodes one of the small subunits in photosystem (PS) II (Kohzuma et al., 2017). The latter is known as a *G* mutant (Nagai, 1921). Recently, the responsible gene related to the *G* locus was identified using genome-wide association analysis (Wang et al., 2018). It is suggested that the green seed coat trait caused by *G*, which encodes a CAAX protease, originated from wild traits, and modern yellow varieties possess a mutant allele *g* against the *G* allele (Wang et al., 2018). There is a homologous gene for *G* in the soybean genome and the genes are functionally redundant in determining green pigmentation in soybean tissues (Liu et al., 2020). However, the relationship between *G* and its homologous genes in terms of the green pigmentation of the seed coat remains unclear. The spatial expression of these genes also remains unknown in seed tissues. In addition, if the geographical distribution of the mutant alleles for these genes is elucidated, we may better understand the establishment of yellow soybean.

Previously, we demonstrated that mature wild soybean accumulates large amounts of β-carotene and chlorophyll in the seed coat (Kanamaru et al., 2006). However, the relationship between chlorophyll accumulation in wild soybean and the *G* locus remains unknown. If the pigmentation in green seed coats is perfectly derived from the chlorophyll accumulation, the chlorophyll pigmentation can be seen. However, dark colors, such as black and brown, in seed coats obscure the appearance of chlorophyll pigmentation. When chlorophyll accumulation is evaluated in the seed coats of dark-colored or wild soybean, it is necessary to isolate and measure the chlorophyll. In this study, to elucidate the molecular mechanism of chlorophyll accumulation in the seed coat of wild soybean, quantitative trait loci (QTL) analysis was performed in recombinant inbred lines (RILs) established from a cross between a wild soybean accession and a soybean breeding line. The relationship between the genotypes for *G* and chlorophyll content was also investigated in the seed coats of wild and cultivated soybeans. In addition, the functional characterization was evaluated in near isogenic lines (NILs) established from a cross between green and yellow soybean. Mutant alleles of *G* and its homologous genes were evaluated in soybean distributed in East Asia and other areas. We concluded that the establishment of yellow color in soybean requires the mutation of *G* but not its homologous genes.

## Materials and Methods

### Plant Materials

We obtained a population of 96 RILs derived from a cross between a wild soybean accession (B01167) and a breeding line (TK780) (Liu et al., 2007) from LegumeBase. An F_8_ generation of the RILs was developed in a greenhouse at Hokkaido University, Japan. The other wild and cultivated soybeans and landraces were provided by LegumeBase^1^ and the Genebank of the National Agriculture and Food Research Organization.^2^

### High Performance Liquid Chromatography Analysis of Chlorophyll

Chlorophyll extraction and high performance liquid chromatography (HPLC) analysis were performed using the method described by Monma et al. (1994) with some modifications. Seed coats or cotyledons were collected from 3–8 seeds and ground using a mortar and pestle with liquid nitrogen. Subsequently, 20 mg of seed coat powder was weighed into a 1.5-mL tube and mixed with 1.0 mL of extraction solution (acetone:ethanol = 1:1) by stirring. After 20 min, the mixture was centrifuged at 15,000 × *g* for 10 min at room temperature. The supernatant was again centrifuged at 15,000 × *g* for 10 min at room temperature. Approximately 300 μL of the resulting upper phase was used for HPLC analysis as described below.

High performance liquid chromatography analysis was performed using Hitachi LaChrom Elite (Hitachi High-Technologies Corp., Tokyo, Japan) with an Inertsil ODS3 column (4.6 × 250 mm, GL Science, Tokyo, Japan). For this, 20 μL of the supernatant was injected onto the column. The mobile phase flow rate was 1.0 mL/min during the entire run, and the column was maintained at 40°C. A linear gradient system was applied using two mobile phases: ethanol and acetonitrile. The gradient was initiated at 25% ethanol (v/v) and then increased to 80% from 0 to 15 min. It was subsequently decreased to 25% ethanol from 15 to 17 min and maintained at 25% for 5 min. The UV-Vis detector (Hitachi, L-2420) was set at 445 nm to quantify chlorophylls. Standard reagents for chlorophyll *a* and *b* were obtained from DHI (Horsholm, Denmark). The content of each type of chlorophyll was determined based on the ratio of the area of the respective peaks to standard chlorophylls.

### Quantitative Trait Loci Analysis

Quantitative trait loci analysis for the chlorophyll content in seed coats was performed using F_8_ plants. Permutation analysis (1,000 times) was performed to determine the genome-wide minimum significant LOD threshold score. Based on the result, QTLs with a LOD score of >2.0 were regarded as effective loci. Initial QTL mapping was performed using the interval mapping method provided in MapQTL 5.0 (Van Ooijen, 2004). Markers flanking the QTLs were considered as cofactors using the MQM method in the same program.

### Genomic DNA Extraction From Leaves or Mature Seeds

Genomic DNA extraction from pieces of leaf (approximately 5 mm × 5 mm) or mature seeds was performed according to the method described by Sugano et al. (2020). The extracted DNA was used for gene cloning, sequencing, and genotyping the *G* and *G-like* (*GL*) loci.

### Total RNA Extraction From Seed Coat and Cotyledon of Immature Seeds

Total RNA was extracted from seed coats or cotyledons of immature seeds using the LiCl precipitation method (Adachi et al., 2021). cDNA was synthesized from the total RNA and was used for gene cloning, sequencing, expression analysis, and vector construction.

### Gene Cloning and DNA Sequencing

PCR fragments amplified using specific primers (Supplementary Table 1) were sequenced directly or after cloning into the pGEM-T-Easy vector (Promega, Madison, United States) using the Big Dye terminator cycle method with an ABI3100 or ABI3130 Genetic Analyzer (Thermo Fisher Scientific, Waltham, United States). DNA sequencing analysis was performed by the Instrumental Analysis Division, Graduate School of Agriculture, Hokkaido University.

### Genotyping of the *G* and *GL* Loci

Genotyping of the *G* and *GL* loci was conducted based on the derived cleaved amplified polymorphic sequence (dCAPS). PCR analysis of the *G* locus was conducted in a 20-μL volume under the following conditions: 35 cycles of 94°C for 30 s, 53°C for 30 s, and 72°C for 20 s, using a specific primer set (Supplementary Table 1). The amplified products were digested with *Dde*I. PCR analysis of the *GL* locus was performed in a 20-μL volume under the following conditions: 35 cycles of 94°C for 30 s, 50°C for 30 s, and 72°C for 20 s, using a specific primer set (Supplementary Table 1). The amplified products for the *G* locus were digested with *Bsp*T104I. The digested products were separated on a 2.5% agarose gel.

### Expression Analysis of *G* and *GL*

Quantitative RT-PCR was performed in a 20-μL volume containing 9.2 μL of diluted cDNA solution, 0.8 μL of each primer (1 μM), and 10 μL of SYBR Premix Ex Taq II (Tli RNaseH Plus) (TaKaRa Bio, Tokyo, Japan). The reaction was performed using a CFX96 Real-Time System (Bio-Rad Laboratories Inc., Tokyo, Japan) under the following conditions: 40 cycles of 95°C for 30 s, 56°C for 30 s, and 72°C for 30 s. The specificity of amplification was verified with a melting curve. The expression levels of *G* and *GL* were normalized to the expression level of the β-tubulin gene (Glyma.08G014200). The gene expression levels were assessed by CAPS analysis using the SNP between *G* and *GL*. The PCR analysis was conducted in a 20-μL volume under the following conditions: 30 or 35 cycles of 94°C for 30 s, 56°C for 30 s, and 72°C for 20 s, using a common primer set for the *G* and *GL* loci (Supplementary Table 1). The amplified products were digested with *Sac*I, and the digested products were separated on a 2.5% agarose gel. The transcript levels of *G*, *g*, and *GL* were evaluated relative to those of the β-tubulin gene.

### Characterization of the Seed Coat in Near Isogenic Lines

The NILs (F_6_ generation) for the *G* locus were developed according to the single seed descent method from an F_2_ population derived from a cross between Tenshindaiseitou and Ichihime. The NILs were grown in an experimental field (N43°04′, E141°21′) at Hokkaido University. The chlorophyll content and weight of the seed coat were evaluated in the immature seeds of each individual 21, 30, 39, 46, 50, and 60 days after the first flowering (DAF). The chlorophyll content was evaluated by HPLC analysis. The weight of the seed coat was expressed as the weight of one seed. Six individuals from each NIL were used for these analyses.

### Evaluation of Photosynthetic Activity in Near Isogenic Lines

The NILs (F_6_ generation) were grown in the greenhouse at Hokkaido University. Six individuals from each NIL were used to evaluate the photosynthetic performance of the leaves. The chlorophyll content was estimated based on the SPAD value using a chlorophyll meter (SPAD-502, Konica Minolta, Tokyo, Japan). The SPAD readings were taken in triplicate from the ninth leaf at 7, 14, 28, 36, 46, 49, and 53 DAF, and the averages were used to represent the SPAD values of each individual. The photosynthetic carbon exchange rate was measured using a portable photosynthesis system (LI-6400, LI-COR, Nebraska, United States) on the ninth leaf of each individual at 17, 28, 36, and 46 DAF. All gas exchange measurements were performed between 8:00 and 12:00 with the leaf chamber settings for CO_2_ concentration of 400 μmol mol^–1^, a photosynthetic photon flux density of 1,500 μmol m^–2^ s^–1^, relative humidity of 60%, and an air temperature of 28°C. Minimal and maximal Chl fluorescence intensities of the ninth leaf were measured at 17, 28, 36, and 46 DAF using a pulse-amplitude modulated chlorophyll fluorometer (Junior PAM, Walz, Effeltrich, Germany) on fully dark-adapted ninth leaves on the night of the gas exchange measurement to determine the maximum quantum yield of PS II (Fv/Fm).

### Germination and Permeability Tests in Near Isogenic Lines

The NILs (F_7_ generations) were grown in an experimental field at Hokkaido University. Six individuals of each NIL were used for the germination and permeability tests. Germination was defined as the point at which the tip of hypocotyl broke through the seed coat. Twenty seeds from each individual were used immediately after harvesting for the germination test. The seeds were placed on a filter paper moistened with sterile water and observed for germination every 4 h. Permeability was assessed by measuring the weight of the seeds before and after immersion in water. Twenty seeds were immersed in sterile water and their weight was measured at intervals of 1 h.

### Vector Construction for Soybean Transformation

We constructed an expression vector for the *G* locus (pGmG-99). *G* was amplified from the cDNA of Tenshindaiseitou using specific primers (Supplementary Table 1). The amplified gene was placed under the control of the cauliflower mosaic virus 35S (CaMV 35S) promoter and the terminator from an *Arabidopsis* (*Arabidopsis thaliana*) heat shock protein gene in the binary vector pRI 201-AN (TaKaRa Bio). The expression unit was inserted into the binary vector pMDC99 (Curtis and Grossniklaus, 2003).

### Soybean Transformation

A soybean variety (Jack), which possesses the ability to undergo somatic embryo induction and regeneration from immature cotyledons (Tomlin et al., 2002), was used for transformation. Biolistic transformation was performed according to the method described by El-Shemy et al. (2004). Tissue culture was conducted under a 16 h light: 8 h dark cycle (photosynthetic photon flux density: 20–50 μmol m^–2^ s^–1^) at 26°C. Transgenic plants were grown in commercial soil (Katakura Chikkarin Co., Tokyo, Japan) at 25°C in a greenhouse isolated for transgenic plants at Hokkaido University.

### Examination of Chlorophyll Degradation in Transgenic Soybean Plants

The extent of chlorophyll degradation was examined in seedlings of control (Jack) and transgenic soybean plants overexpressing *G*. One of the unfolded primary leaves of the seedlings was covered with aluminum foil to shade it. The degree of chlorophyll degradation was evaluated by the intensity of green color of the primary leaves 7 days after shading.

### Assay to Determine the Subcellular Localization of the G and GL Proteins

Plasmids expressing *GFP* fused to *G* or *GL* under the control of the *Arabidopsis UBQ10* promoter were constructed as follows. The CaMV 35S promoter, multiple cloning site, and *NOS* terminator were amplified by PCR using the pJ4 vector (Fukazawa et al., 2021) as a template. *GFP* was amplified by PCR using pMOE-GFP (Takeo and Ito, 2017) as a template. These DNA fragments were subcloned into the *Not*I and *Asc*I sites of pENTR (Thermo Fisher Scientific), yielding pENTR-35S-GFP. The coding sequences of *G* and *GL* were amplified by PCR with soybean cDNA as a template and cloned into pENTR-35S-GFP. The *Arabidopsis UBQ10* promoter was amplified by PCR and replaced with the CaMV 35S promoter of pENTR-35S-GFP carrying *G* or *GL* using NEBuilder (New England Biolabs, Ipswich, United States). The primers used for PCR are shown in Supplementary Table 1. G-GFP and GL-GFP fusion proteins were transiently expressed in *Arabidopsis* mesophyll protoplasts as described by Ito and Fukazawa (2021). GFP fluorescence was detected using the LSM 5 Pascal confocal microscope (Carl Zeiss, Oberkochen, Germany) and ZEN 2009 software (Carl Zeiss).

### Statistical Analysis

Tests of significance among means of data was performed using Student’s *t*-test. *P*-values < 0.05 were considered to indicate statistical significance.

## Results

### Quantitative Trait Loci Analysis of the Chlorophyll Content in Seed Coats of Mature Seeds in Recombinant Inbred Lines Established From a Cross Between Wild and Cultivated Soybeans

**FIGURE 2:**
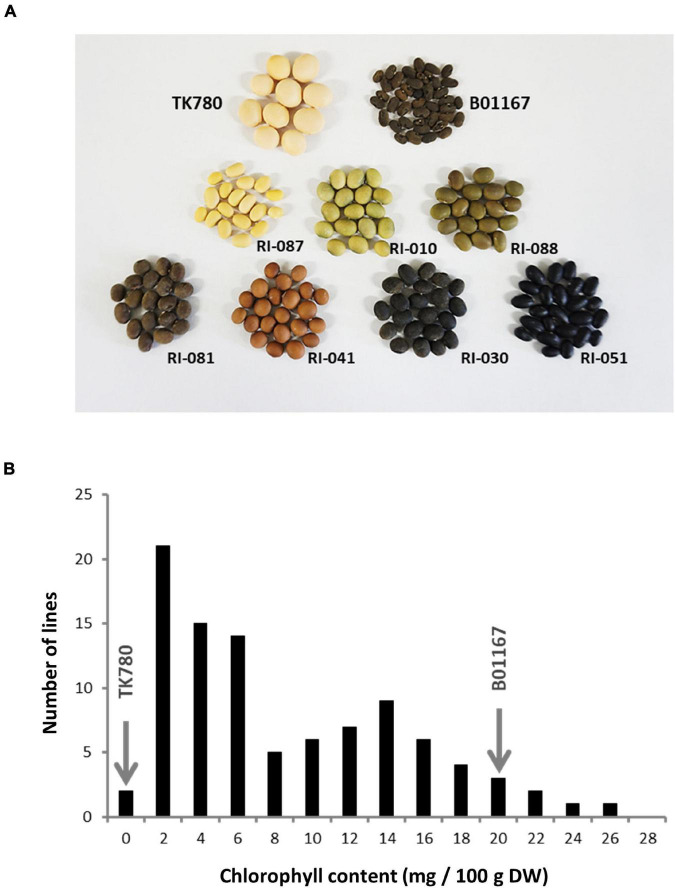
Colors and chlorophyll contents of the seed coat in representative RILs generated from a cross between cultivated and wild soybeans and parental plants. **(A)** Representative RILs generated from a cross between a wild soybean accession (B01167) and a breeding line (TK780). RILs (F_8_ generation) and parental plants were grown in the greenhouse at Hokkaido University. The seeds in the middle and bottom rows show the seed coat colors of representative RILs. The number at the bottom right of each RIL seed indicates the line number of the RIL. **(B)** Frequency distribution of the chlorophyll content in the seed coats of RILs and parental plants. The arrows denote the chlorophyll content of parental plants.

The RILs established from a cross between a soybean breeding line (TK780) and a wild soybean accession (B01167) exhibited various colors, including yellow, black, green, dark brown, and reddish brown, in their seed coats (Figure 2A). The chlorophyll content in RIL seed coats was evaluated by HPLC. The concentration of chlorophyll in the seed coat of the RILs ranged from 0.00 to 25.18 mg/100 g dry weight (DW; Figure 2B), with TK780 and B01167 producing it at concentrations of 0.07 ± 0.05 and 19.3 ± 1.0 mg/100 g DW in their seed coats, respectively. High levels of chlorophyll were also found in black or brown seed coats. Following QTL analysis of the chlorophyll content in the seed coats of the RILs, three loci (*qSCC1*, *qSCC5*, and *qSCC6*) were identified (Table 1). The first, *qSCC1*, which was detected on chromosome 1, had the largest effect and explained 40.9% of the phenotype (Table 1 and Supplementary Figure 1). Localization using molecular markers revealed that *qSCC1* was closely linked to the *G* locus. We concluded that the gene responsible for *qSSC1* corresponded to *G* because we found the same single nucleotide change between B01167 and TK780 as the SNP that was identified in the *G* locus by Wang et al. (2018). We designed a dCAPS marker to detect the SNP at the *G* locus, and alleles of B01167 and TK780 at the *G* locus were distinguished as “*G*” and “*g*,” respectively. The *G* and *g* alleles clearly explained the differences in the distribution of chlorophyll in the seed coats among the RILs (Supplementary Figure 2).

**TABLE 1:** QTLs associated with chlorophyll contents in mature seed coats assessed by multiple QTL mapping.

Chromosome no.	Nearest marker^a^	LOD score^b^	PVE (%)^c^	Additive effect^d^
Chr 1	AGA/CAC310	13.8	40.9	−4.0
Chr 5	Satt545	2.1	9.9	−1.9
Chr 6	AGG/CGC380	2.5	11.5	−2.1

*^a^AGA/CAC310 and AGG/CGC380 denote amplified fragment length polymorphism markers and Satt545 denotes an simple sequence repeat marker.*

*^b^LOD = logarithm of odds, the peak of the LOD value in the QTL range.*

*^c^PVE = percentage of phenotypic variance explaining the chlorophyll content.*

*^d^Positive values of the additive effect = increased effect for the QTL was caused by TK780.*

*QTL: quantitative trait loci.*

### Relationship Between the Chlorophyll Contents of Seed Coats and Genotypes of the *G* Locus in Soybean Germplasm

**FIGURE 3:**
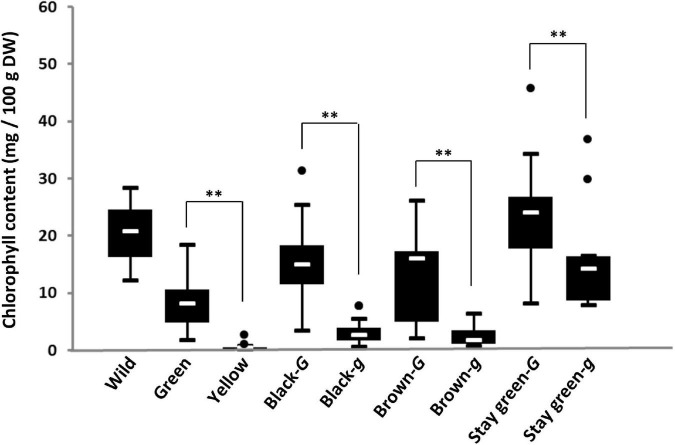
Box-plots of the chlorophyll content in the seed coats of soybean germplasm. Eight soybean groups were evaluated: wild soybean (Wild), green seed coat (Green), yellow seed coat (Yellow), black seed coat with the *G* allele (Black-*G*), black seed coat with the *g* allele (Black-*g*), brown seed coat with the *G* allele (Brown-*G*), brown seed coat with the *g* allele (Brown-*g*), green cotyledon with the *G* allele (stay-green-*G*), and green cotyledon with the *g* allele (stay-green-*g*). The seed coat color and genotypes for the *G* locus of soybean germplasm used in this study are listed in Supplementary Table 2. White lines in black boxes indicate mean values. ^**^ denotes a significant difference at the 1% level.

To examine the relationship between genotypes of the *G* locus and chlorophyll content in the seed coat, genotyping of the *G* locus and measurement of the chlorophyll content of the seed coat were performed in soybean germplasm lines. All 16 accessions of wild soybean used in this study possessed the *G* allele and contained a large amount of chlorophyll in their seed coats (12.4–38.3 mg/100 g DW) (Figure 3). Genotyping of the *G* locus revealed that all yellow and green seed coat soybeans surveyed in this study possessed *g* and *G* alleles, respectively (Supplementary Table 2). There was a significant difference in the chlorophyll content of the seed coat between green and yellow soybeans (Figure 3). The other soybean resources were classified into three groups: brown seed coat, black seed coat, and green cotyledon (Figure 3). These groups were further categorized based on the presence of the two alleles *G* and *g* because both alleles were detected among brown and black seed coat and green cotyledon soybeans (Supplementary Table 2). For each seed coat color, soybeans with the *G* allele accumulated significantly more chlorophyll in the seed coat than those with the *g* allele (Figure 3).

### Development and Characterization of Near Isogenic Lines for the *G* Locus

**FIGURE 4:**
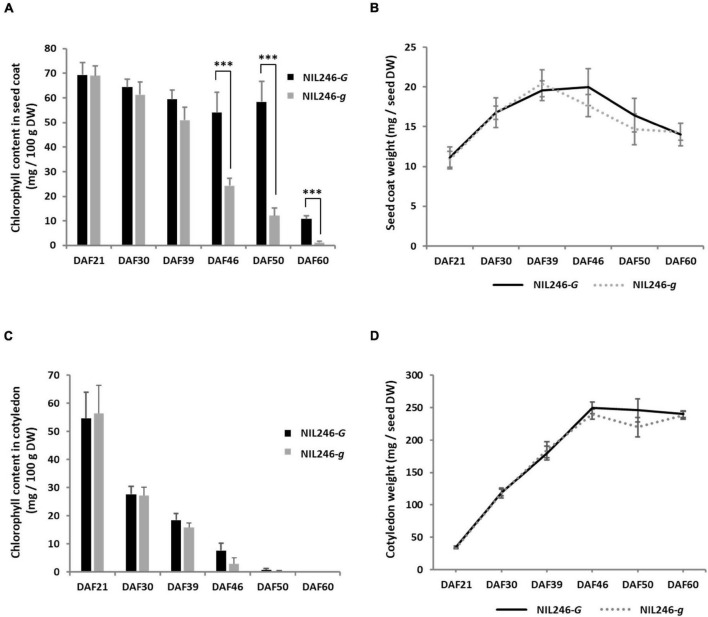
Characterization of seed coats and cotyledons in NIL246s (yellow cotyledons). **(A)** Chlorophyll content of the seed coat. ^***^ indicates the significant differences between NIL246-*G* and NIL246-*g* at the 0.1% level. **(B)** Seed coat weight per seed. **(C)** Chlorophyll content of the cotyledon. **(D)** Cotyledon weight per seed. All data shown are the mean ± SD of six individuals.

To confirm the pleiotropic effects of the *G* locus, NILs were generated. A crossing population was generated between stay-green (Tenshindaiseitou) and yellow (Ichihime) soybean varieties and subsequently self-pollinated (Supplementary Figure 3). The color of the seed coats and cotyledons was segregated in the progenies because Tenshindaiseitou possessed the *d*_1_ and *d*_2_ alleles for stay-green properties and the *G* allele for the *G* locus (Supplementary Figure 3 and Supplementary Table 2). The NILs (F_5_) were developed using the single seed descent method from the F_2_ population. Each NIL for the *G* locus was developed under the genetic background of yellow (*D_1_D_1_D_2_D_2_*) or green cotyledons (*d_1_d_1_d_2_d_2_*) in the F_5_ generation. In a yellow cotyledon-NIL (NIL246), there was no difference in the chlorophyll content in the seed coat between the *G* (NIL246-*G*; *D_1_D_1_D_2_D_2_GG*) and *g* (NIL246-*g*; *D_1_D_1_D_2_D_2_gg*) alleles up to 39 DAF, but a significant difference was detected at >46 DAF (Figure 4A). However, the seed coat weight per seed was the same between NIL246-*G* and NIL246-*g* (Figure 4B). There was also no difference between NIL246-*G* and NIL246-*g* in terms of chlorophyll content and cotyledon weight at each seed developmental stage (Figures 4C,D).

**FIGURE 5:**
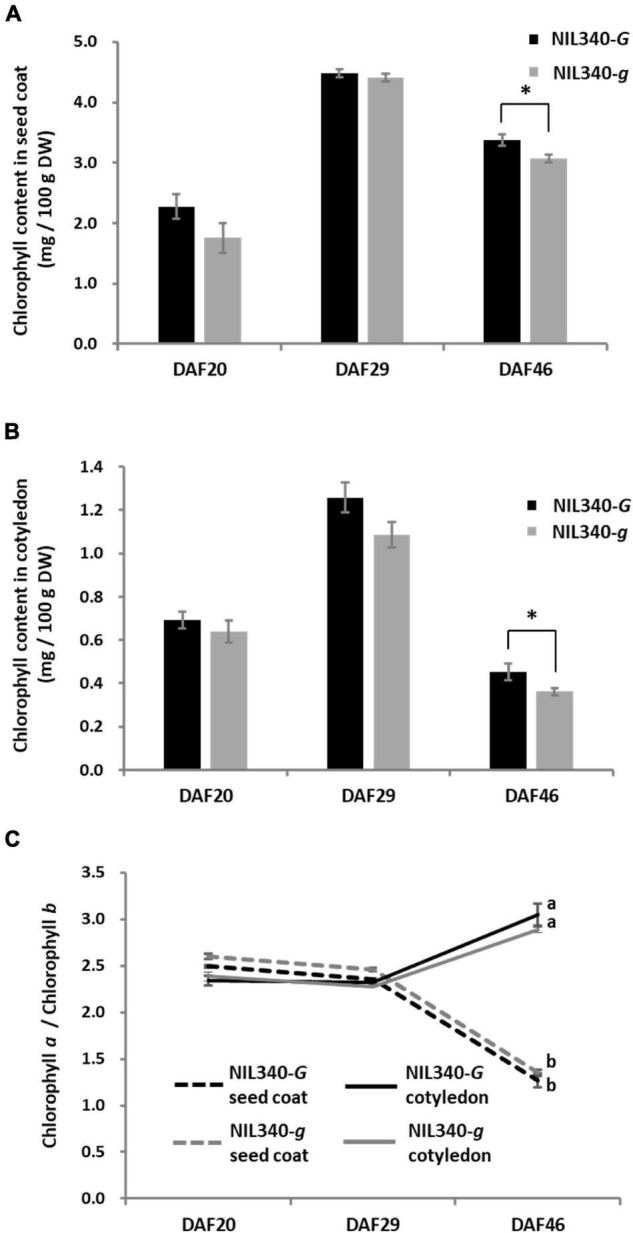
Characterization of seed coats and cotyledons in NIL340s (green cotyledons). **(A)** Chlorophyll content of the seed coat. * indicates the significant differences between NIL340-*G* and NIL340-*g* at the 5% level. **(B)** Chlorophyll content of the cotyledon. * indicates the significant differences between NIL340-*G* and NIL340-*g* at the 5% level. **(C)** Composition (*a*/*b*) of the chlorophyll *a* and *b* content in seed coats and cotyledons. Different letters at DAF46 denote significant differences (*P* < 0.01). All data shown are the mean ± SD of four individuals.

In a green cotyledon-NIL (NIL340), no differences were found in the chlorophyll content between the *G* (NIL340-*G*; *d_1_d_1_d_2_d_2_GG*) and *g* (NIL340-*g*; *d_1_d_1_d_2_d_2_gg*) alleles up to 29 DAF (Figures 5A,B). However, in mature seeds at >46 DAF, the presence of the *G* allele increased the chlorophyll content in the seed coat and cotyledon (Figures 5A,B and Supplementary Figures 4A,B). Although there was no difference in the composition of chlorophyll (chlorophyll *a*/*b*) between NIL340-*G* and NIL340-*g*, distinct differences were observed between the seed coat and the cotyledon (Figure 5C). Up to 29 DAF, the chlorophyll composition was the same between the seed coat and the cotyledon, but at 46 DAF, the ratio between chlorophyll *a*/*b* decreased in the seed coat and increased in the cotyledon (Figure 5C).

The effect of the *G* locus on the photosynthetic activity in leaves was evaluated in NIL246s. The SPAD value, photosynthetic carbon exchange rate, and maximum quantum yield of PS II (Fv/Fm value) were compared between the ninth leaves of NIL246-*G* and NIL246-*g* (Supplementary Figure 5). The ninth leaves were fully expanded at 17 DAF and had completely yellowed by 53 DAF. The SPAD, carbon exchange rate, and Fv/Fm values of both NILs markedly decreased between 28 and 46 DAF (Supplementary Figure 5). There was no significant difference in photosynthetic activity between NIL246-*G* and NIL246-*g* (Supplementary Figure 5). The physiological characteristics of mature seeds (germination and permeability ratios) were examined in the NILs (NIL246-*G*, NIL246-*g*, NIL340-*G*, and NIL340-*g*). Although the germination time was almost the same between yellow cotyledon-NILs, a slight difference was observed between the green cotyledon-NILs (Supplementary Figure 6). However, the germination ratios of NIL246s and NIL340s reached approximately 100% at 52 and 72 h after sowing, respectively (Supplementary Figure 6). The permeability of the seeds was also evaluated in the NILs. The weight of all seeds reached approximately 250% compared with that of dried seeds 30 h after soaking (Supplementary Figure 7). The permeability of NIL246-*g* was found to be slightly higher (evidenced by faster water permeation) than that of NIL246-*G* (Supplementary Figure 7).

### Characterization of Transgenic Soybean Overexpressing *G*

**FIGURE 6:**
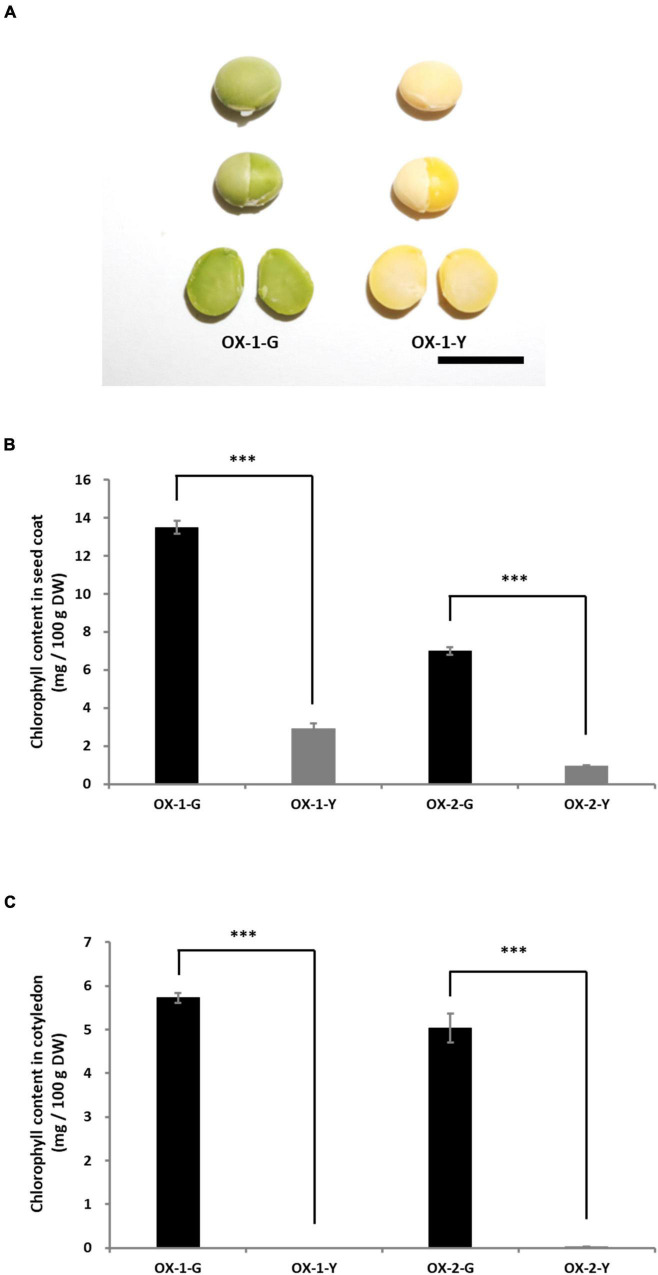
Characterization of transgenic plants overexpressing *G*. **(A)** Appearances of seed and cotyledons of transgenic lines. OX-1-G and OX-2-G indicate green seed coat lines. OX-1-Y and OX-2-Y indicate yellow seed coat lines. In the T_1_ progeny, the green and yellow seed coat traits are genetically segregated. Scale bar = 1 cm. The chlorophyll content in the seed coat **(B)** and cotyledons **(C)**. Significant differences were observed in the chlorophyll content of seed coats and cotyledons between -G and -Y lines. ^***^ indicates significant differences at the 0.1% level.

To clarify the function of the *G* allele, *G* (LC649881) was cloned from the cDNA of Tenshindaiseitou. *G* was expressed under the control of the CaMV 35S promoter. Two independent T_0_ transgenic plants (OX-1 and OX-2) were obtained through the biolistic transformation of soybean somatic embryos. The seed coats and cotyledons were segregated into green and yellow colors in the T_1_ generations of both OX-1 and OX-2. Therefore, fixed lines for seed coat color were developed in the T_2_ generation, namely OX-1-G (green seed line), OX-1-Y (yellow seed line), OX-2-G, and OX-2-Y. PCR analysis using the set of specific primers for the transgene revealed that the transgene was removed by genetic segregation in OX-1-Y and OX-2-Y. Seeds harboring the transgene exhibited green color in both seed coat and cotyledon tissues (Figure 6A). However, there was no difference between OX-1-Y and the control seed (Jack) in terms of the appearance of the mature seeds (Supplementary Figure 8). HPLC analysis revealed that the overexpression of *G* resulted in chlorophyll accumulation in both seed coat and cotyledon tissues (Figures 6B,C).

The effect of the overexpression of *G* was also evaluated in the leaf tissue of transgenic soybean plants. We examined the extent of chlorophyll accumulation in primary leaves of Jack, yellow seed lines, and green seed lines. The difference in the degree of chlorophyll accumulation due to the overexpression of *G* could not be visually confirmed in the primary leaves (Supplementary Figure 9). Therefore, we exposed primary leaves to dark conditions to promote chlorophyll degradation. The shaded leaves of OX-1-G and OX-2-G showed a weak stay-green trait, whereas those of Jack, OX-1-Y, and OX-2-Y showed yellow color (Supplementary Figure 9).

### Characterization of *G* and *GL*

*GL* (Glyma.11G043400) is a homologue of *G* and is located on chromosome 11. In examining the amino acid sequences of the G, g, and GL proteins, the sequence of the latter was found to be 32 residues longer at the N-terminal than that of the G protein (Supplementary Figure 10). However, the putative amino acid sequence of the g protein was 44 residues shorter than that of the G protein at the C-terminal region (Supplementary Figure 10). The similarity of the amino acid sequences, excluding the 32 residues at the N-terminal, was 97.6% between the G and GL proteins (Supplementary Figure 10). This similarity was higher than that (90.0%) between the g and G proteins (Supplementary Figure 10). To examine the subcellular localization of the G and GL proteins, expression vectors carrying fusion proteins of G or GL with GFP under the control of the *Arabidopsis UBQ10* promoter were constructed (Supplementary Figure 11A). When the G-GFP and GL-GFP fusion proteins were transiently expressed in *Arabidopsis* mesophyll protoplasts, GFP fluorescence signals from G-GFP and GL-GFP were colocalized with the autofluorescence of chloroplasts, suggesting that the G and GL proteins are localized in chloroplasts (Supplementary Figure 11B).

To examine the expression characteristics of *G* and *GL*, gene expression analysis was performed in NIL246s generated by a cross between Tenshindaiseitou and Ichihime. The sequence of the *GL* gene in Ichihime (yellow soybean) corresponded perfectly to that of Tenshindaiseitou (stay-green soybean). Quantitative RT-PCR analysis revealed that the expression of *G* and *g* in the seed coat was the same between NIL246-*G* and NIL246-*g* (Supplementary Figure 12A). *G* and *GL* were expressed both in the seed coat and cotyledons (Supplementary Figure 12B). The expression of *G* and *GL* in young leaves tended to be much higher than that in the seed coat in NIL246-*G* (Supplementary Figures 12C,D). Because different regions of *G* and *GL* were amplified by quantitative RT-PCR, differences in their expression could not be evaluated. To compare the expression levels of *G* and *GL*, a common primer set was designed (Supplementary Figure 13A), and the expression levels were distinguished by the presence or absence of restriction enzyme recognition sites in the amplified products (Supplementary Figures 13A,B). We evaluated the amplification efficiency using the common primer set before the expression analysis. The results of a CAPS analysis of genomic DNA showed no difference between *G* and *GL* (Supplementary Figure 13C). Therefore, we judged that there was no difference in the amplification efficiency of PCR using this common primer set for both *G* and *GL*. In the seed coat, the expression level of *G* was slightly higher than that of *GL* (Supplementary Figure 13D). However, the expression of both genes in the cotyledons was almost the same (Supplementary Figure 13D).

### Distribution of *G*, *GL*, and Their Mutant Alleles in Soybean Germplasm

**FIGURE 7:**
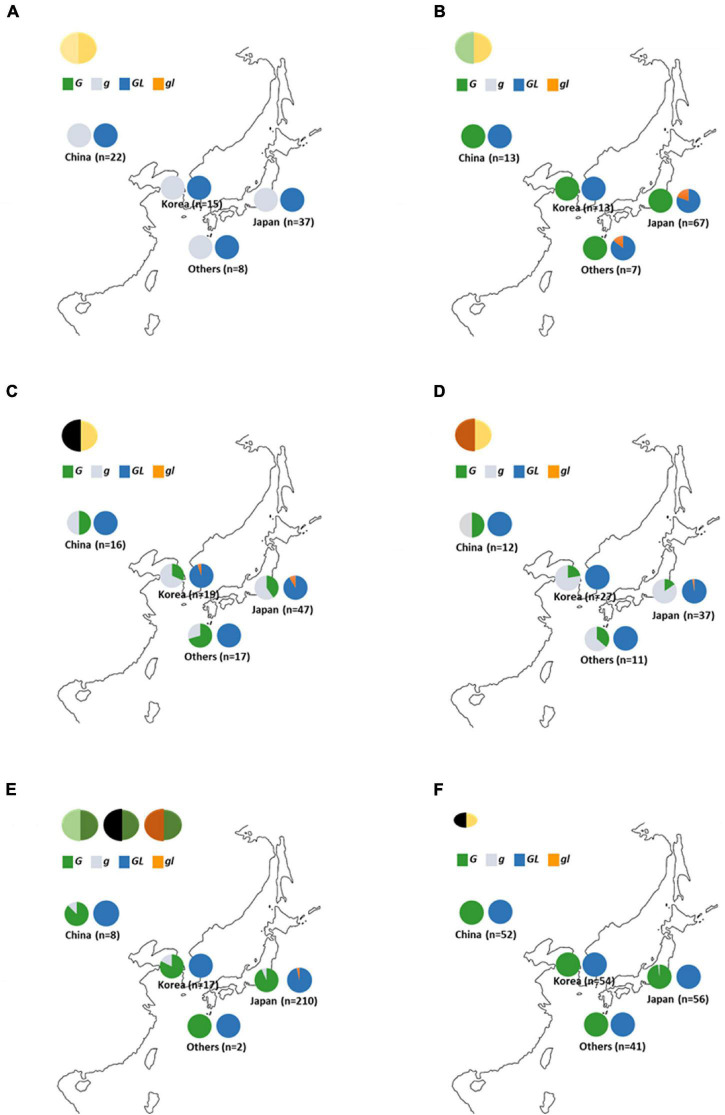
Distribution of *G* and *GL* alleles in soybean germplasm. **(A)** Yellow seed coat soybeans. **(B)** Green seed coat soybeans. **(C)** Black seed coat soybeans. **(D)** Brown seed coat soybeans **(E)** Green cotyledon soybeans**. (F)** Wild soybeans. “Others” include soybean germplasm from outside China, Korea, and Japan. Green, gray, navy, and orange in graphs indicate the ratio of the *G*, *g*, *GL*, and *gl* alleles, respectively.

Variations of *GL* have been detected among early-yellowing mutants (Liu et al., 2020). In this study, the same early-yellowing trait was observed in the segregation population developed from green seed coat (Wase-edamame) and yellow seed coat breeding lines (TH152), and the sequences of the *G* and *GL* loci were analyzed in these lines. A single nucleotide deletion with a frameshift mutation was detected at the *GL* locus of Wase-edamame (Supplementary Figure 14A). We named this mutant allele *gl* (Supplementary Figure 13A; LC649882). This mutation caused the stop codon of the putative gl protein to appear earlier than that of the putative GL protein (Supplementary Figure 14B). The dCAPS markers were designed for the *G* and *GL* loci to examine the distribution of these mutant alleles among cultivated and wild soybean germplasm (Supplementary Figure 15 and Supplementary Tables 3, 4). All 82 yellow soybean lines used in this study possessed the *g* and *GL* alleles (Figure 7A). All 100 green seed coat soybean lines examined in this study possessed the *G* allele (Figure 7B). However, the *gl* allele was detected in 14 green seed coat soybean lines (Figure 7B and Supplementary Table 4). Furthermore, 13 of the 14 accessions were derived from Japanese soybean germplasm (Figure 7B and Supplementary Table 4). In black seed coat lines, 54 of 99 harbored the *g* allele (Supplementary Table 3). The *g* allele was widely distributed among black seed coat soybeans in China, Korea, Japan, and other regions (Figure 7C and Supplementary Table 3), whereas the *gl* allele was detected in only five lines (Figure 7C). Four of the five accessions were Japanese (Figure 7C and Supplementary Table 4). A total of 88 brown seed coat lines were examined and 66 accessions had the *G* genotype for the *G* locus (Figure 7D and Supplementary Table 3). Among the brown soybeans, only one accession, which originated in Japan, contained the *gl* allele (Figure 7D and Supplementary Table 3). In total, 15 of 237 green cotyledon soybeans exhibited the *g* genotype for the *G* locus and 8 accessions had the *gl* allele (Figure 7E and Supplementary Table 3). All mutant alleles for the *GL* locus were found in Japanese accessions (Figure 7E and Supplementary Table 4). A total of 204 wild soybean accessions used in this study originated from China (52), Korea (54), Japan (57), and other areas (41). Most of the wild soybeans contained the *G* and *GL* alleles (Figure 7F). Only one Japanese accession (B090092) had the *g* allele at the *G* locus (Figure 7F and Supplementary Table 3). This Japanese wild soybean accession had a clearly bigger seed size compared with other wild soybeans (Supplementary Figure 16). No soybean line among the landraces and breeding lines investigated in this study contained both the *g* and *gl* alleles.

## Discussion

The *G* and *GL* loci encode a CAAX protease (Wang et al., 2018; Liu et al., 2020), but there is a large difference at the N-terminal region between the G and GL proteins (Supplementary Figure 10). The GL protein has an N-terminal region that is 32 residues longer than that of the G protein (Supplementary Figure 10). In general, the amino acid sequence of the N-terminal region is closely associated with the subcellular localization of the protein because it tends to include the transit peptides required for localization to specific subcellular organelles. Examination of the subcellular localizations of the G and GL proteins revealed that both were localized in plastids (Supplementary Figure 11B), indicating that differences in the amino acid sequence in the N-terminal region had no effect on the subcellular localization of these proteins. In *Arabidopsis*, two orthologs (BCM1 and BCM2) for the G protein play highly conserved roles in the chlorophyll metabolic pathway (Wang et al., 2020). The two homologous genes have redundant functions in different tissues and developmental stages (Wang et al., 2020). Loss of function of both *G* and *GL* has been shown to result in yellowing in the leaves during the early growth stage in soybean (Liu et al., 2020). However, *G* and *GL* differ significantly in their roles associated with the accumulation of chlorophyll in the seed coat. Among the green seed coat soybean resources examined in this study, the mutant allele was observed only at the *GL* locus, with no mutant allele identified at the *G* locus (Figures 3, 7B). Compared with the *g* allele, the presence of the *G* allele was advantageous for the accumulation of chlorophyll in the seed coat (Figure 3). Gene expression analyses also revealed that the expression of *G* and *GL* in the seed coat was comparable (Supplementary Figures 12, 13). There are several differences in the amino acid sequence between the G and GL proteins in addition those seen in the N-terminal region (Supplementary Figure 10), indicating that the variations in the amino acid sequences rather than the expression levels of *G* and *GL* might contribute to differences in chlorophyll accumulation in the seed coat.

Chlorophyll accumulation in the cotyledons of mature seeds was not detected, although the expression of *G* was found in the cotyledons of immature seeds in NIL246-*G*, which has yellow cotyledons (Supplementary Figure 12). Surprisingly, overexpression of *G* resulted in the accumulation of chlorophyll not only in the seed coat but also in the mature cotyledons of transgenic soybean plants (Figures 6B,C and Supplementary Figure 8). This indicates that high expression of *G* results in chlorophyll accumulation that exceeds chlorophyll metabolism in soybean seeds. On the other hand, overexpression of *G* resulted in a weak stay-green trait in the leaf tissue (Supplementary Figure 9). The difference in the degree of the stay-green trait observed between tissues might be due to differences in the degree of chlorophyll metabolism between seed and leaf tissues. Analysis of the NILs for the *G* locus revealed that those containing the *G* allele exhibited significantly higher chlorophyll contents in the seed coats and cotyledons than those harboring the *g* allele in the *d_1_d_2_* genetic background (Figures 5A,B and Supplementary Figure 5). The *Arabidopsis* G-ortholog BCM1 is known to interact with Genomes Uncoupled 4 (GUN4) and Mg-Proto methyltransferase (CHLM), which are important in chlorophyll biosynthesis (Wang et al., 2020). Zhang et al. (2020) also demonstrated that this protein behaves as a magnesium transport protein to maintain magnesium homeostasis in the chloroplast. These findings indicate that G-ortholog is strongly involved in chlorophyll biosynthesis. Although chlorophyll biosynthesis might be enhanced in NIL340-*G*, no increase in chlorophyll levels was observed during early seed filling in this line compared with NIL340-*g* (Figures 5A,B). These results suggested that the G protein might repress the SGR-independent degradation of chlorophyll. Interestingly, although changes in the chlorophyll content in the seed coat and cotyledons showed similar patterns of increases and decreases during seed ripening in NIL340, the ratio of chlorophyll *a* and *b* (Chl *a*/Chl *b*) differed greatly between the seed coat and cotyledons at 46 DAF (Figure 5C). Two photosystems, PSI and PSII, on thylakoid membranes, such as the grana core vesicles and stroma lamella vesicles, play an important role in the light reaction during photosynthesis in higher plants (Anderson and Melis, 1983; Danielsson et al., 2004). PSI and PSII are accompanied by antenna proteins comprising the light-harvesting complex (LHC). These LHC proteins bind chlorophyll *a* and *b*, although the ratio of these is different among the LHC proteins (Green et al., 1991). The SGR protein is associated with chlorophyll degradation by inducing LHCII disassembly through direct reaction (Park et al., 2007). The degradation of chlorophyll during senescence has been suggested to be different between the seed coat and cotyledon.

Most wild accessions examined in this study possessed the *G* allele at the *G* locus (Figure 7F), indicating that the *G* allele is derived from wild soybean and the accumulation of chlorophyll in the seed coat is a wild trait. Wang et al. (2018) stated that the *G* locus is involved in seed dormancy, and transgenic soybean seeds expressing *G* have a slower germination rate than non-transgenic plants before breaking dormancy. In this study, no effect of dormancy was observed in the experiment using yellow cotyledon-NILs (246-*G* and 246-*g*) (Supplementary Figure 7A). Differences reported in previous studies may be due to differences in the genetic backgrounds of the examined soybeans. However, NIL340-*g* germinated at a slightly faster rate than 340-*G* under the genetic background of *d_1_d_2_* stay-green (Supplementary Figure 6B). The over-accumulation of free chlorophyll, which possesses photosensitizing properties and causes a burst of reactive oxygen species upon light exposure, caused severe photodamage in the maturing seeds of stay-green *Arabidopsis* mutants (Li et al., 2017). Similar photodamage was observed in the *d_1_d_2_* soybean mutant (Li et al., 2017). The slight delay in germination observed in 340-*G* might be also due to photodamage. To evaluate the pleiotropy of *G*, we examined the permeability of the NIL seeds (Supplementary Figure 7). The permeability of NIL246-*g* was slightly higher (evidenced by faster water permeation) than that of NIL246-*G* (Supplementary Figure 7A). The QTL for physical seed dormancy in soybean, which is caused by hardseededness, has been detected on chromosome 2 and the responsible genes have been isolated (Jang et al., 2015; Sun et al., 2015). Although the *G* locus does not contribute to a trait like hardseededness, the accumulation of chlorophyll in the seed coat may change its structure and slightly affect seed permeability. The green seed coat trait has been found in several legumes, including pea (*Pisum sativum*), azuki bean (*Vigna angularis*), and chickpea (*Cicer arietinum*) (Segev et al., 2010; Marles et al., 2013; Horiuchi et al., 2015). Therefore, the accumulation of chlorophyll in the seed coat might be common in legumes. If the wild trait of chlorophyll accumulation in the seed coat were to cause any disadvantages to the development of cultivated soybeans, the distribution of the *G* allele would be strongly limited. However, the *G* allele was widely distributed in various soybeans, including those with black and brown seed coats and green cotyledons (Figures 7C–E). These results indicated that the presence of the *G* allele is not likely to be a major obstacle to soybean domestication, except for the green pigmentation of the seed coat.

The seed size of cultivated soybeans used in this study was much larger than that of wild soybean germplasm regardless of the *G* locus genotypes. On the other hand, only one wild accession (B09002) from Japan possessed the *g* allele at the *G* locus (Figure 7F and Supplementary Table 3) and produced clearly larger seeds compared with other wild soybeans (Supplementary Figure 16). Kuroda et al. (2006) demonstrated the introgression from cultivated soybeans to wild soybeans using a variation analysis based on microsatellite markers. It was suggested that the gene flow from cultivated soybeans to wild accessions increased the seed size and introduced the *g* allele to B09002. However, the only trace of gene flow from cultivated soybean was the gigantism of seed shown in B09002. The geographical distribution of the mutation at the *GL* locus was very limited. Most of the *gl* alleles were identified in Japan (Figure 7), indicating that the *gl* allele might have evolved recently in Japan. All of the *gl* mutants examined in this study had the *G* allele at the *G* locus. Wang et al. (2020) mentioned that the loss of function of both *G* and *GL* causes yellowing of the soybean plant. Therefore, the G protein is thought to complement the function of the GL protein in *gl* mutants. This indicated that the loss of function of both the *G* and *GL* alleles would severely inhibit soybean growth, but the loss of one of these alleles can be compensated for by the other. However, as only the *G* allele is essential for green pigmentation in the seed coat, we concluded that the mutation of the *G* locus alone had been essential to establishing yellow soybean, which is a major current soybean breeding line.

## Data Availability Statement

The datasets presented in this study can be found in online repositories. The names of the repository/repositories and accession number(s) can be found below: https://www.ncbi.nlm.nih.gov/genbank/, LC649881; https://www.ncbi.nlm.nih.gov/genbank/, LC649882.

## Author Contributions

YTo, MK, JA, and TY designed the study. YTo, TK, HN, AH, HS, YTa, JA, and TY developed the cross segregating populations. YTo, TK, AK, TN, and TY analyzed the soybean germplasm and the NILs. YTo, TK, and MI produced and analyzed the transgenic soybean plants. HY, TI, and MK analyzed the subcellular localization of the proteins. TY wrote the manuscript. All authors contributed to the article and approved the submitted version.

## Conflict of Interest

The authors declare that the research was conducted in the absence of any commercial or financial relationships that could be construed as a potential conflict of interest.

## Publisher’s Note

All claims expressed in this article are solely those of the authors and do not necessarily represent those of their affiliated organizations, or those of the publisher, the editors and the reviewers. Any product that may be evaluated in this article, or claim that may be made by its manufacturer, is not guaranteed or endorsed by the publisher.

## Footnotes

1
https://www.legumebase.brc.miyazaki-u.ac.jp/top.jsp

2
https://www.gene.affrc.go.jp/index_en.php
