# Supplementary material for: Functional Divergence of G and Its Homologous Genes for Green Pigmentation in Soybean Seeds
Source: Front Plant Sci. 2022 Jan 5;12:796981. doi: 10.3389/fpls.2021.796981 (PMC8766641; doi:10.3389/fpls.2021.796981)
Supplement: Supplementary file 1 [file Data_Sheet_1.docx]

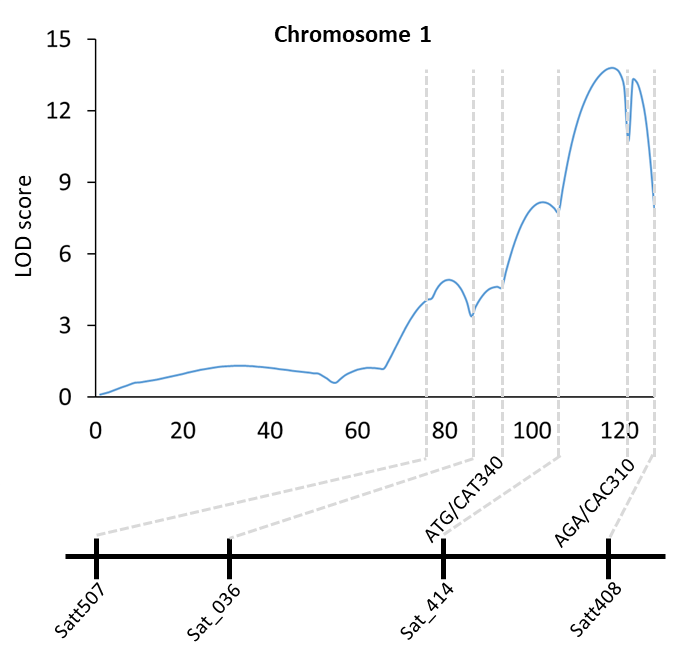


**Supplementary Figure S1.** QTL for chlorophyll content in seed coat on chromosome 1. The vertical line indicates the LOD-value profile from MQM mapping of chlorophyll content in seed coat on chromosome 1. The horizontal line corresponds genetic distances (Kosambi cM). Dashed lines show positions of molecular markers. Information on molecular markers can be obtained from a previous report (Liu et al. 2007).


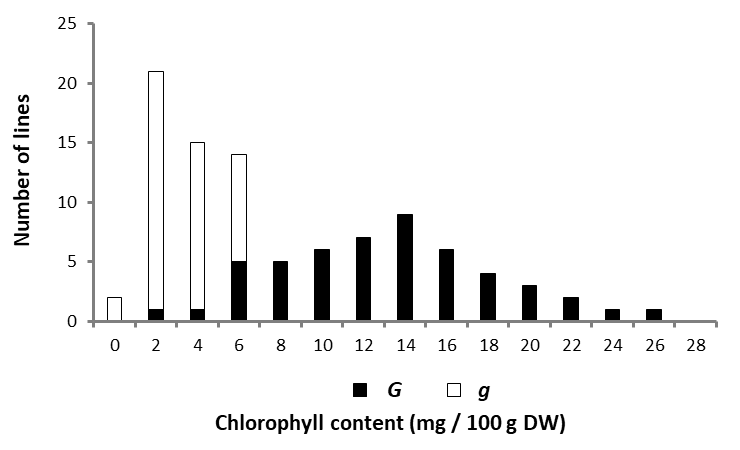


**Supplementary Figure S2.** Frequency distribution of chlorophyll content of seed coat and genotypes of *G* locus in F_8_ population. Black and white bars represent *G* and *g* alleles for the *G* locus, respectively. Genotypes for the *G* locus were determined by the dCAPS analysis using the specific primer set (Supplementary Table S1).


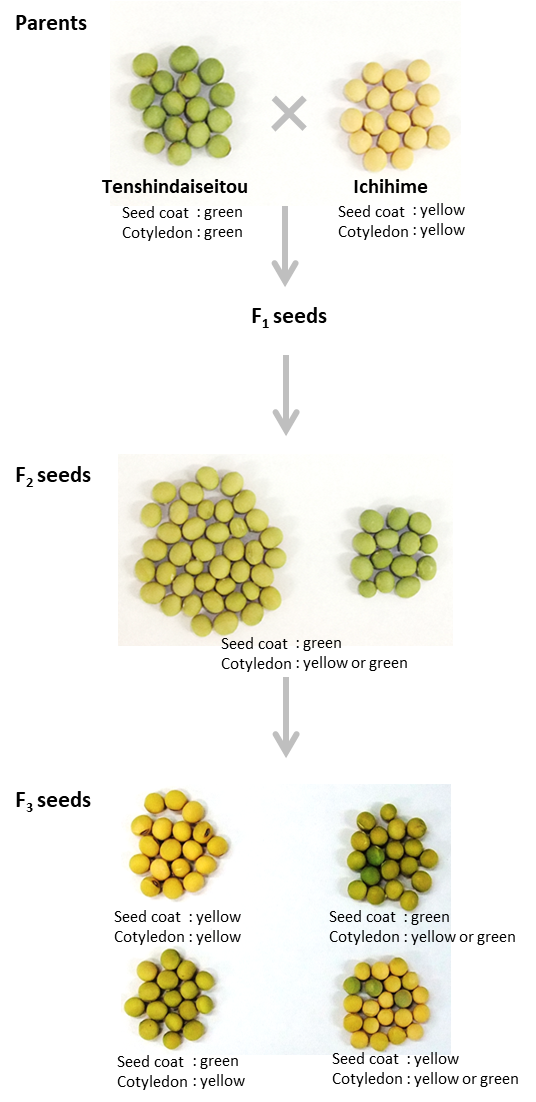


**Supplementary Figure S3.** Seed coat and cotyledon colors in progenies between Tenshindaiseitou (stay green soybean) and Ichihime (yellow soybean). Segregation of seed coat and cotyledon colors is observed in F_3_ seeds.


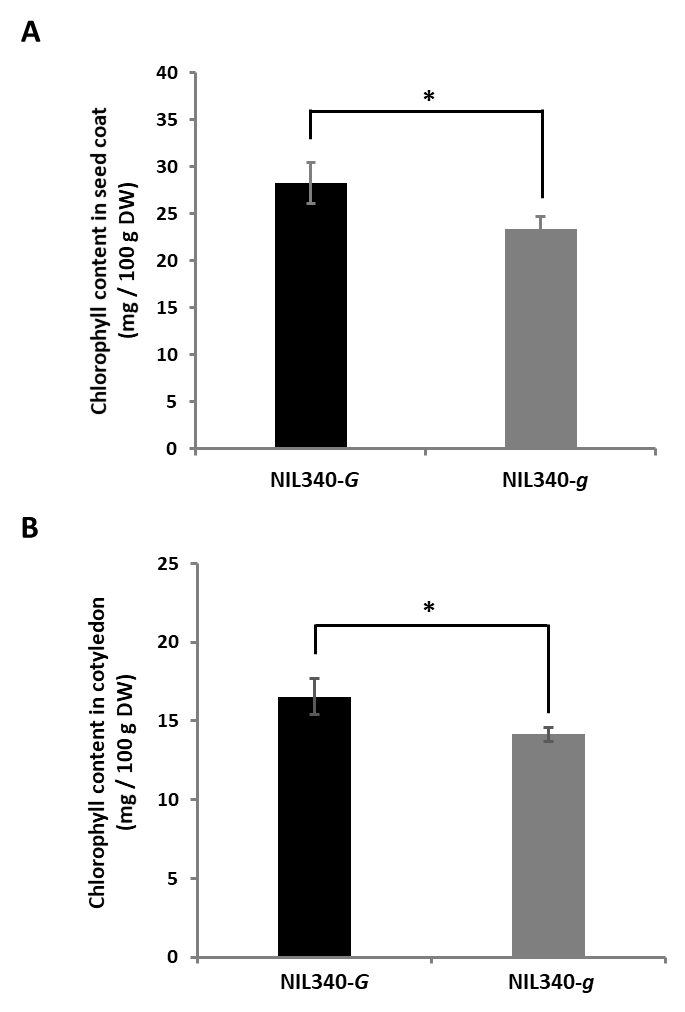


**Supplementary Figure S4.** Chlorophyll content of seed coats and cotyledons of mature seeds in NIL340s. (A) Chlorophyll content of seed coat. (B) Chlorophyll content of cotyledon. Data shown are mean ± SD of four individuals. * indicate the significant differences between NIL340-*G* and NIL340-*g* at the 5% level.


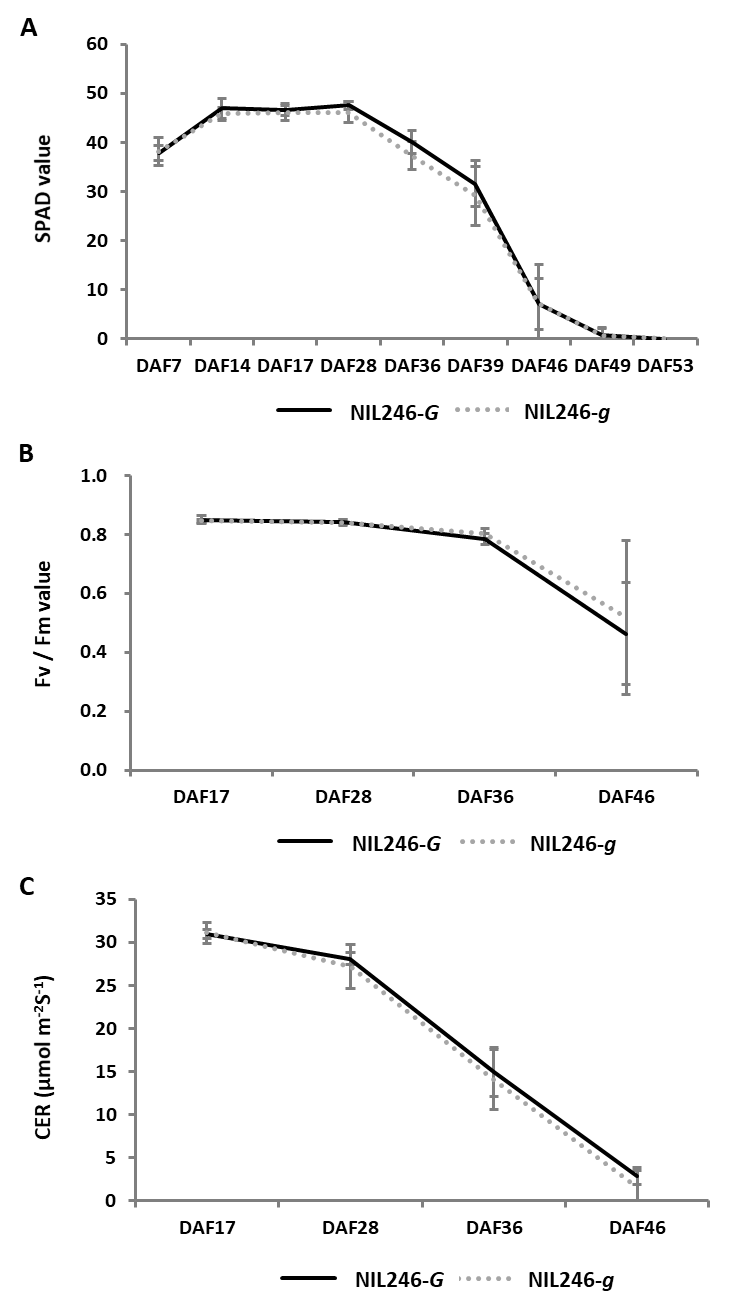


**Supplementary Figure S5.** Photosynthetic activity in leaves of NIL246s. (A) SPAD value. (B) Maximum quantum yield of photosystem II (Fv/Fm). (C) Photosynthetic carbon exchange rate (CER). Data shown are mean ± SD of six individuals. Data were evaluated as significantly different between NIL246-*G* and NIL246-*g* if *P*-values were less than 0.05 (Student’s *t* test). No significant differences were found between NIL246-*G* and NIL246-*g*.


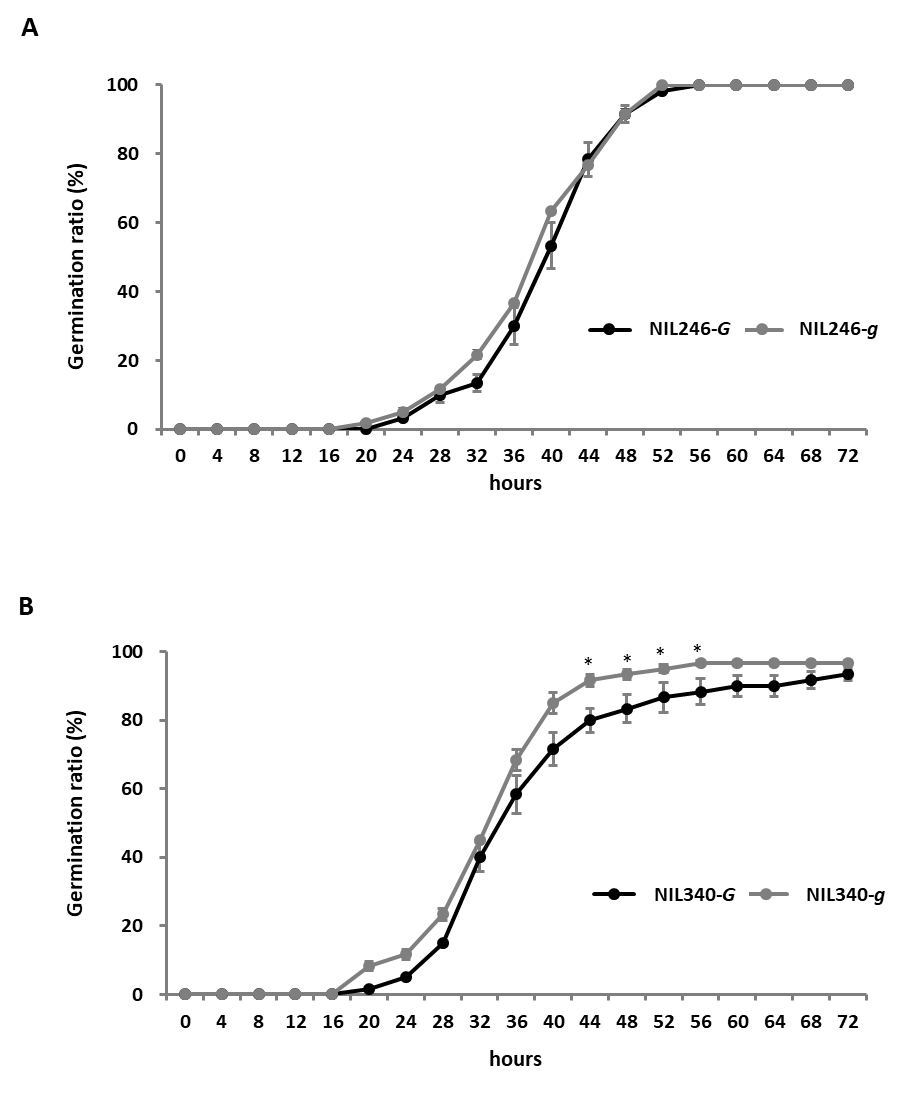


**Supplementary Figure S6.** Germination ratio of NILs. (A) Germination of yellow cotyledon NIL (NIL246s). (B) Germination ratio of green cotyledon NIL (NIL340s). Three individuals of each NIL were used for the germination test. Twenty seeds of each individual, immediately after harvesting, were used for the germination test. The seeds were placed on the filter paper moistened with sterile water and was observed for the germination every 4 hours. Data shown are mean ± SD of three independent experiments. * indicate significant differences between the *G* and *g* alleles at the 5% level.


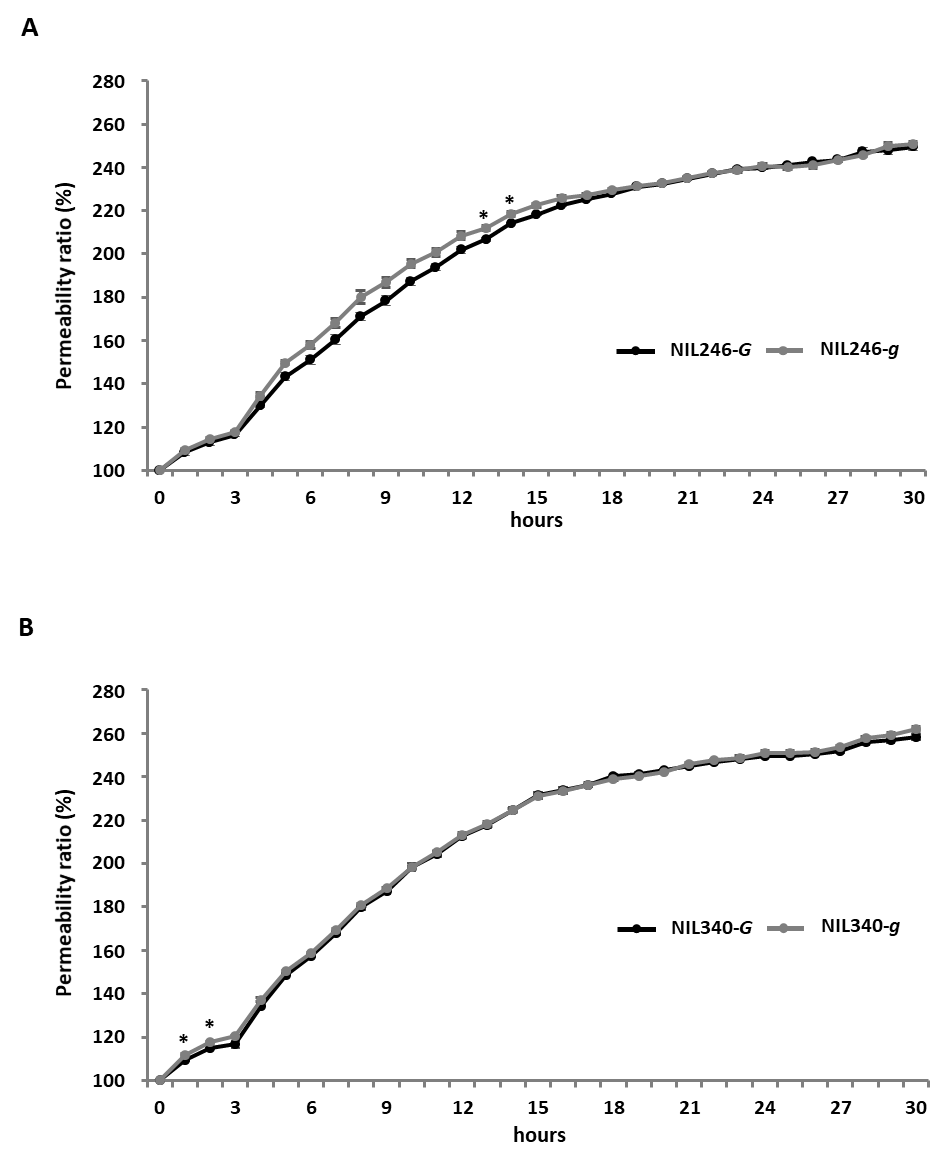


**Supplementary Figure S7.** Permeability ratio of NILs. (A) Permeability ratio of yellow cotyledon NIL (NIL246s). (B) Permeability ratio of green cotyledon NIL (NIL340s). The permeability ratio was calculated as 100% of the seed weight before the permeability test. Three individuals of each NIL were used for the permeability test. Twenty seeds of each individual, immediately after harvesting, were used for the permeability test. The seeds were placed on the filter paper moistened with sterile water and was observed for the germination every hour. Data shown are mean ± SD of three independent experiments. * indicate significant differences between the *G* and *g* alleles at the 5% level.


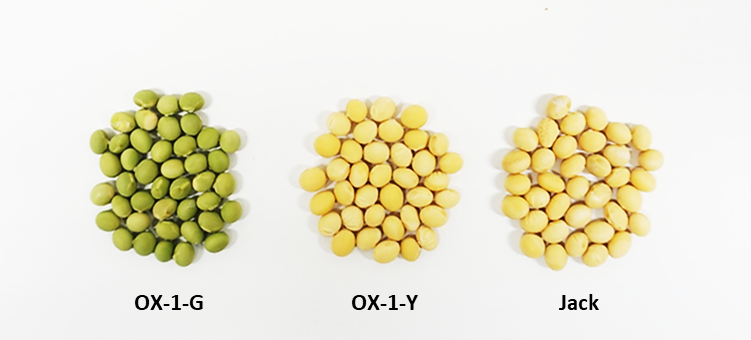


**Supplementary Figure S8.** Mature seeds (T_2_ seed) of transgenic and control plants. OX-1-G and OX-1-Y were originated from an OX-1 T_0_ transgenic plant. Mature seeds of OX-1-G, OX-1-Y, and Jack did not show significant differences in size or shape.


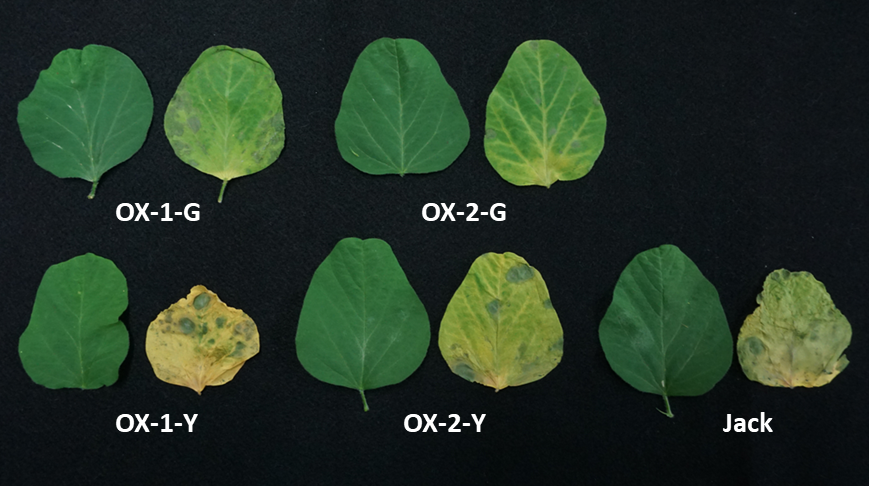


**Supplementary Figure S9.** Representative image in chlorophyll-degradation examination of primary leaves of transgenic (T_3_ generation) and control plants. Left and right leaves indicate untreated primary leaves and primary leaves 7 days after dark incubation, respectively.


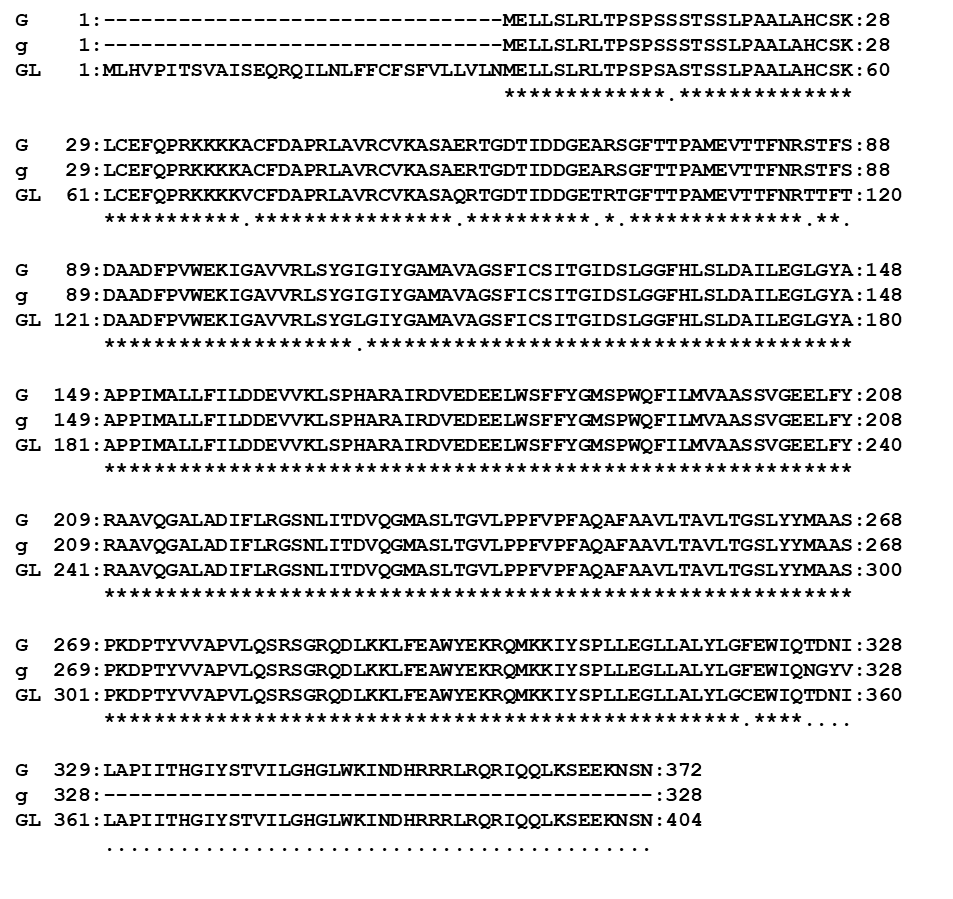


**Supplementary Figure S10.** Sequence similarity of G, g, and GL proteins. The numbers on the left and right sides indicate the number of amino acid residues from the start codon. * denote that the amino acid residues are the same in the three proteins.


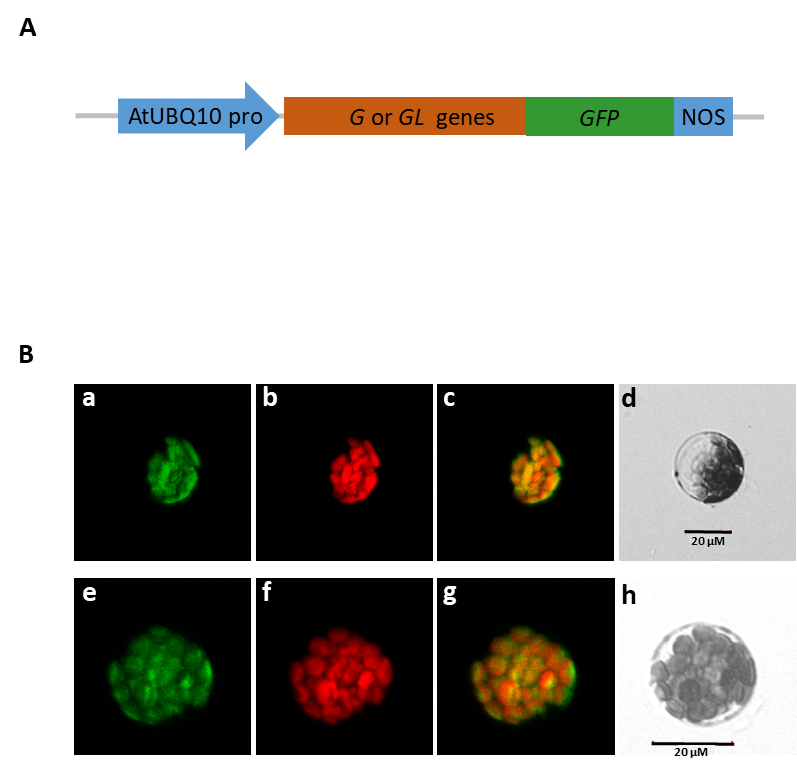


**Supplementary Figure S11.** Subcellular localization of the G-GFP and GL-GFP fusion proteins. (A) Structure of constructs for the transient expression assay of the fusion proteins. AtUBQ10 promoter and NOS indicate the promoter of Arabidopsis ubiquitin 10 gene and terminater of Agrobacterium nopaline synthase gene (B) Confocal microscopy images of Arabidopsis mesophyll protoplasts expressing the fusion proteins. Upper and bottom panels show protoplasts expressing G-GFP and GL-GFP fusion proteins, respectively. GFP Fluorescence (a, e), Autofluorescence of chloroplasts (b, f), Marge (c, g), Visible light (d, h). Scale bars denote 20 μm.


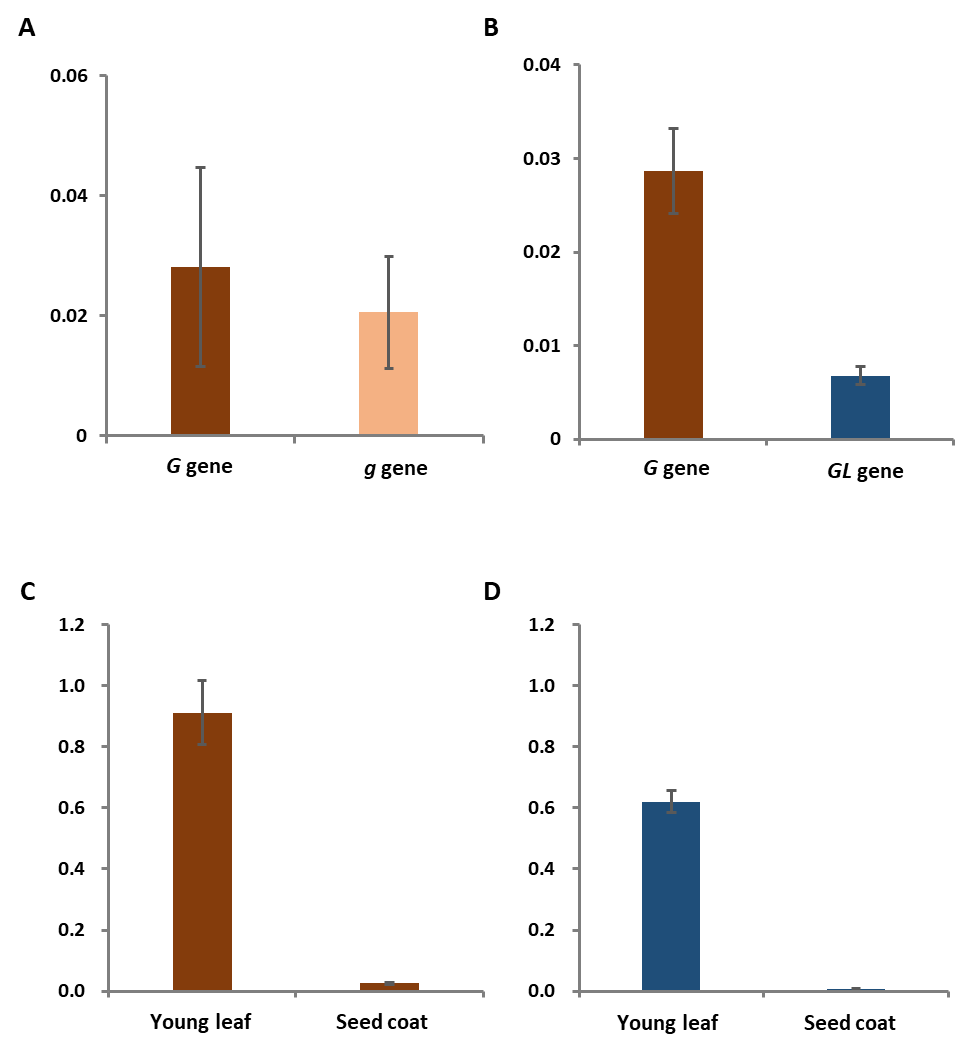


**Supplementary Figure S12.** Expression level of *G*, *g*, and *GL* genes by quantitative RT-PCR in NIL246-*G* and NIL246-*g*. Relative expression level determined by normalizing the PCR threshold cycle number of *G*, *g*, and *GL* genes to that of the β-tubulin gene (Glyma.08G014200). Data are means ± SD of four biological replicates. (A) Expression levels of *G* in NIL246-*G* and *g* in NIL246-*g* genes in seed coat (R6 developmental stage). Common primer set was used for expression analysis. (B) Expression levels of *G* and *GL* genes in NIL246-*G*. Different primer sets were used for expression analysis. (C) Expression level of *G* gene in young leaf (juvenile leaf before full development at 3 days after the first flowering) and seed coat in NIL246-*G*. (D) Expression level of *GL* gene in young leaf and seed coat in NIL246-*G*.


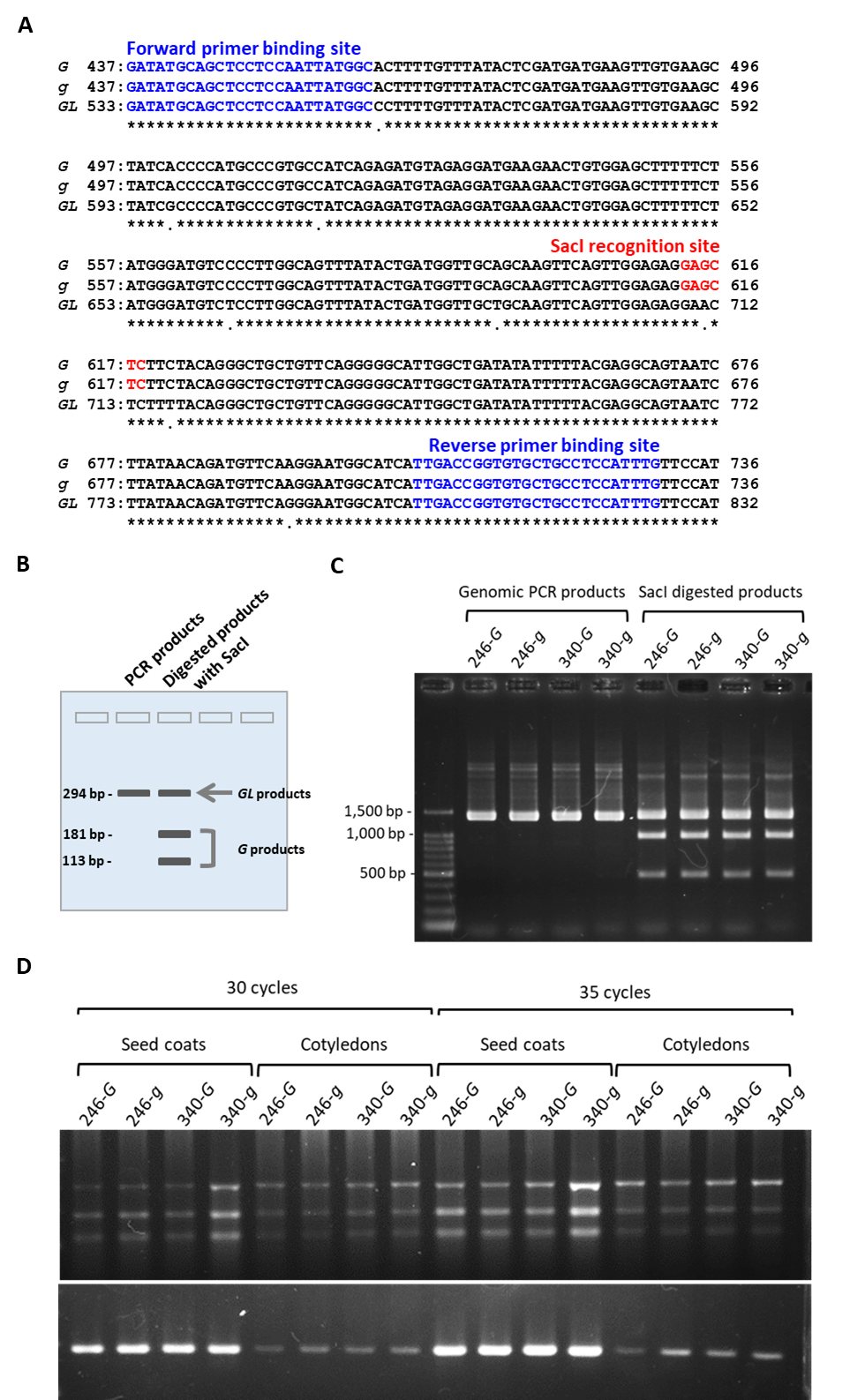


**Supplementary Figure S13.** Expression level of *G*, *g*, and *GL* genes by semi-quantitative RT-PCR in NIL246-*G* NIL246-*g*, NIL340-*G*, and NIL340-*g*. (A) Amplified regions of *G*, *g*, and *GL* genes. The numbers on the left and right sides indicate the number of nucleotides from the start codon in each putative cDNA. * denote the nucleotides are the same in all three genes. Red-colored sequences show the recognition site of SacI restriction enzyme. (B) Schematic diagram of electrophoresis of amplified products and cleaved products by SacI of *G* and *GL* genes. (C) Genomic PCR products and its products digested with SacI. (D) Expression level of *G*, *g*, and *GL* genes by semi-quantitative RT-PCR. Upper and bottom panels indicate electrophoretic images of amplified products and cleaved products by SacI of *G*, *g*, and *GL* genes, and β-tubulin gene, respectively. All DNA fragments are stained with ethidium bromide.


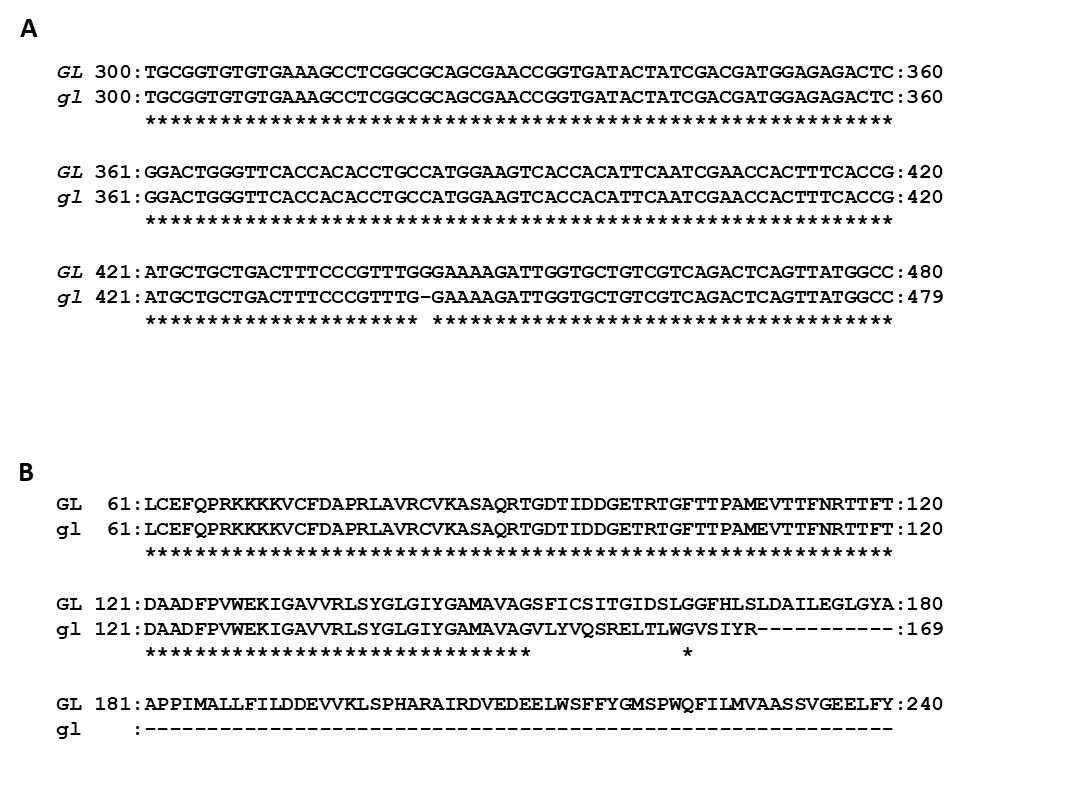


**Supplementary Figure S14.** Sequences of the *GL* and *gl* alleles. (A) Nucleotide sequences of putative cDNA in the *GL* and *gl* alleles. The numbers on the left and right sides indicate the number of nucleotides from the start codon in each putative cDNA. * indicate the nucleotides are the same in the two alleles. (B) Amino acid sequences of putative protein of the GL and gl. The numbers on the left and right sides indicate the number of amino acid residues from the start codon. * denote the amino acid residues are the same between the GL and gl proteins.


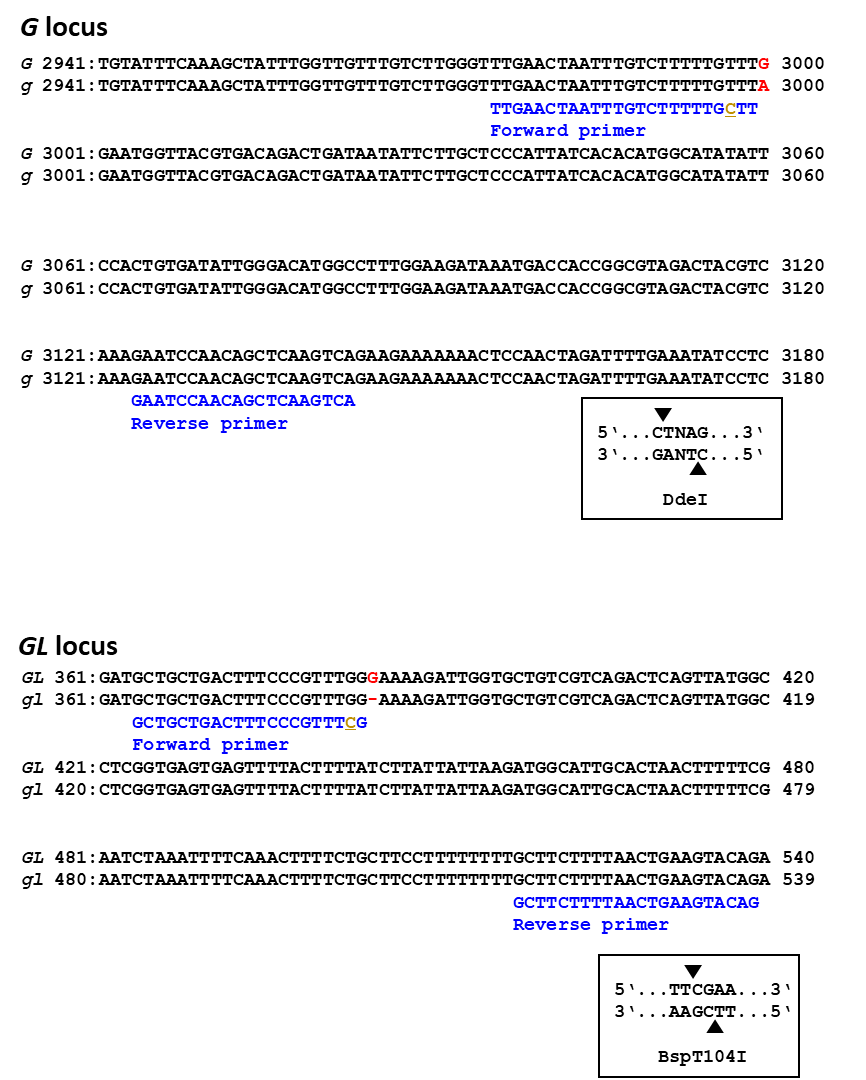


**Supplementary Figure S15.** Molecular (dCAPS) markers of *G* and *GL* loci. Red-colored nucleotides indicate SNP in each locus. Gold-colored nucleotides in primer sequences denote artificial base substitutions in design of dCAPS markers. The numbers on the left and right sides indicate the number of nucleotides from the start codon in each putative cDNA. The restriction enzymes used for dCAPS analysis and their recognition sites are shown in the bottom right frame of each panel.


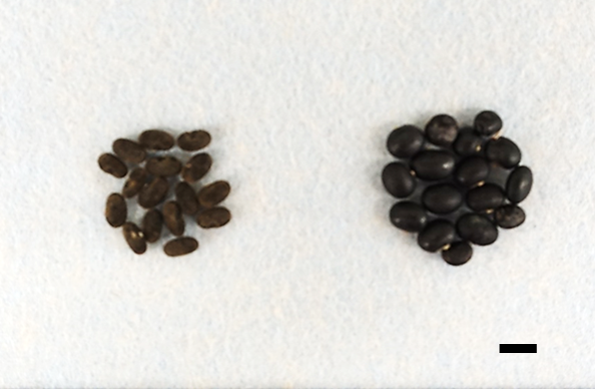


**Supplementary Figure S16.** Morphological characters of wild soybean seeds. Left and right indicate B01167 and B09002 originated from Japan. B09002 showed bigger seeds than other wild soybeans and shiny seed coat. Scale bar denotes 5 mm.

**Supplemental Table S1** Primer sequences used for cloning, sequencing, vector construction, and genotyping analyses.

|  | | |
| --- | --- | --- |
| Primer name | Primer sequence (5'-3') | Use of amplicon |
| G-cDNA_F | GAGACACAGACACAAATAATAAGAG | Gene cloning and sequencing analyses, and vector construction |
| G-cDNA_R^a^ | gcgagctccgGCATATTCTGTATGTACGAGACC | Gene cloning and sequencing analyses, and vector construction |
| G-seq1 | TTGCGGTGCGGTGTGTGAAG | Sequencing analysis |
| G-seq2 | TAGATGCCATTTTGGAAGGGC | Sequencing analysis |
| G-seq3 | CCTACTTATGTTGTTGCACCTG | Sequencing analysis |
| GL_F1 | GATGATGATGATGATGTTGTCGTCGT | Gene cloning and sequencing analyses |
| GL_F2 | CTGTCGTCAGACTCAGTTATGGCC | Gene cloning and sequencing analyses |
| GL_F3 | CCCGTGATTGCTGGGAATATATATAG | Gene cloning and sequencing analyses |
| GL_F4 | GAAAGAATATCACTCATGCTGTATTTGG | Gene cloning and sequencing analyses |
| GL_F5 | GAATCAATTCACATCTTTCTTGTTTACAGT | Gene cloning and sequencing analyses |
| GL_R1 | GATAAAAGTAAAACTCACTCACCGAGG | Gene cloning and sequencing analyses |
| GL_R2 | GTCAGCTCATTAATTAGAAGCTTTCAATTGA | Gene cloning and sequencing analyses |
| GL_R3 | CCACTGAACAAGTTTGAACTGAACC | Gene cloning and sequencing analyses |
| GL_R4 | CCCTAAACACCAACACAACCTACTGAG | Gene cloning and sequencing analyses |
| GL_R5 | GCAAGAATATTATCAGTCTGTCACGTAGT | Gene cloning and sequencing analyses |
| G-SNP_F | TTGAACTAATTTGTCTTTTTGCTT | dCAPS analysis for SNP detection |
| G-SNP_R | TGACTTGAGCTGTTGGATTC | dCAPS analysis for SNP detection |
| GL-SNP_F | GCTGCTGACTTTCCCGTTTCG | dCAPS analysis for SNP detection |
| GL-SNP_R | CTGTACTTCAGTTAAAAGAAGC | dCAPS analysis for SNP detection |
| G-ex_F | CGGATAAACAACCAGGCGTAG | Gene expression analysis |
| G-ex_R | CATGAAACCAACCCAATGGA | Gene expression analysis |
| GL-ex_F | CCCATTATCACACACGGAATATAC | Gene expression analysis |
| GLex_R | CTCCACGACTGATTTTGGTTA | Gene expression analysis |
| G-GL-ex_F | GATATGCAGCTCCTCCAATTATGGC | Gene expression analysis |
| G-GL-ex_R | CAAATGGAGGCAGCACACCGGTCAA | Gene expression analysis |
| G-qPCR_F | CTCCCATTATCCACATGGC | Gene expression analysis |
| G-qPCR_R | CCAAAATCAGTCATGGACATG | Gene expression analysis |
| GL-qPCR_F | CCCATTATCACACACGGAATATAC | Gene expression analysis |
| GL-qPCR_R | TAACCAAAATCAGTCGTGGAG | Gene expression analysis |
| Tublin1404F | GAGAAGAGTATCCGGATAGG | Gene expression analysis |
| Tublin1588R | GAGCTTGAGTGTTCGGAAAC | Gene expression analysis |
| SGR1_F | GCCAATTAGGGCAACCATACCAC | *SGR1* locus genotyping |
| SGR1_R | CTATTTCCGTGGGTCTTGCGTATC | *SGR1* locus genotyping |
| SGR2-commom_F | TGATACGAAACACCCACTACGA | *SGR2* locus genotyping |
| SGR2-commom_R | GACTATCTCATCTCATCTCTGAATGC | *SGR2* locus genotyping |
| SGR2-insert_R | TTGCTACTGCTATTTCGTTATTTAATTCAAGACTG | *SGR2* locus genotyping |

^a^ Sequences in lower letters indicate the linker sequences for vector construction.
